# CCDC61/VFL3 Is a Paralog of SAS6 and Promotes Ciliary Functions

**DOI:** 10.1016/j.str.2020.04.010

**Published:** 2020-06-02

**Authors:** Takashi Ochi, Valentina Quarantotti, Huawen Lin, Jerome Jullien, Ivan Rosa e Silva, Francesco Boselli, Deepak D. Barnabas, Christopher M. Johnson, Stephen H. McLaughlin, Stefan M.V. Freund, Andrew N. Blackford, Yuu Kimata, Raymond E. Goldstein, Stephen P. Jackson, Tom L. Blundell, Susan K. Dutcher, Fanni Gergely, Mark van Breugel

**Affiliations:** 1 MRC Laboratory of Molecular Biology, Cambridge Biomedical Campus, Francis Crick Avenue, Cambridge CB2 0QH, UK; 2 Cancer Research UK Cambridge Institute, University of Cambridge, Li Ka Shing Centre, Robinson Way, Cambridge CB2 0RE, UK; 3 Department of Genetics, Washington University School of Medicine, 4523 Clayton Avenue, St Louis, MO 63110, USA; 4 Wellcome Trust/Cancer Research UK Gurdon Institute, University of Cambridge, Cambridge CB2 1QN, UK; 5 Department of Zoology, University of Cambridge, Downing Street, Cambridge CB2 3EJ, UK; 6 CRTI, INSERM, UNIV Nantes, Nantes, France; 7 DAMTP, Centre for Mathematical Sciences, Wilberforce Road, Cambridge CB3 0WA, UK; 8 Department of Oncology, MRC Weatherall Institute of Molecular Medicine, University of Oxford, John Radcliffe Hospital, Oxford OX3 9DS, UK; 9 Cancer Research UK and Medical Research Council Oxford Institute for Radiation Oncology, University of Oxford, Oxford OX3 7DQ, UK; 10 Department of Genetics, University of Cambridge, Cambridge CB4 1AR, UK; 11 School of Life Science and Technology, ShanghaiTech University, Shanghai 201210, China; 12 Department of Biochemistry, University of Cambridge, 80 Tennis Court Road, Cambridge CB2 1GA, UK

**Keywords:** centrosome, cilia, centriole, basal body, structural biology, CCDC61, SAS6, XRCC4, *Chlamydomonas*, *microtubule*, *VFL3*

## Abstract

Centrioles are cylindrical assemblies whose peripheral microtubule array displays a 9-fold rotational symmetry that is established by the scaffolding protein SAS6. Centriole symmetry can be broken by centriole-associated structures, such as the striated fibers in *Chlamydomonas* that are important for ciliary function. The conserved protein CCDC61/VFL3 is involved in this process, but its exact role is unclear. Here, we show that CCDC61 is a paralog of SAS6. Crystal structures of CCDC61 demonstrate that it contains two homodimerization interfaces that are similar to those found in SAS6, but result in the formation of linear filaments rather than rings. Furthermore, we show that CCDC61 binds microtubules and that residues involved in CCDC61 microtubule binding are important for ciliary function in *Chlamydomonas*. Together, our findings suggest that CCDC61 and SAS6 functionally diverged from a common ancestor while retaining the ability to scaffold the assembly of basal body-associated structures or centrioles, respectively.

## Highlights

• CCDC61 is a paralog of SAS6, XRCC4, XLF, and PAXX
• CCDC61 can form protofilaments with a 3-fold screw axis *in vitro*
• CCDC61 binds to microtubules mainly via its coiled-coil domain
• Microtubule binding of CCDC61 is important for its function in *Chlamydomonas*

## Graphical Abstract

**Figure:**
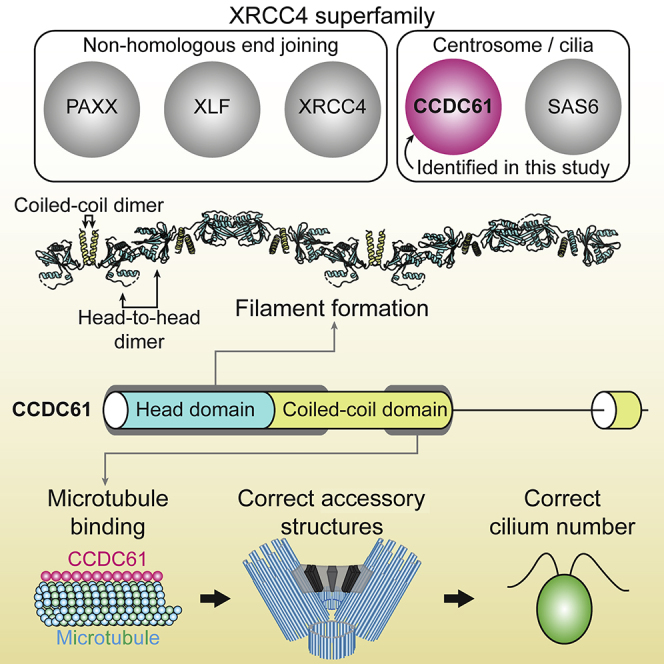

## Teaser

Members of the XRCC4 superfamily of proteins have scaffolding functions in DNA repair or centriole formation. Ochi et al*.* describe the centrosomal protein CCDC61 as a member of this superfamily. CCDC61 oligomerizes and also binds to microtubules which plays a role in establishing accessory structures of centrioles in *Chlamydomonas*.

## Introduction

Centrosomes are among the largest protein assemblies found in animal cells. They function primarily in the organization of the microtubule cytoskeleton and frequently constitute the dominant cellular microtubule organizing center. Due to this function, centrosomes play an important role in ensuring faithful cell division (Nigg and Raff, 2009). Centrosomes are also involved in other critical cellular processes, such as the formation of functional immunological synapses (Stinchcombe and Griffiths, 2014), the organization of actin (Farina et al., 2016), and intracellular signaling (Arquint et al., 2014).

Centrosomes consist of a pair of barrel-shaped centrioles that are surrounded by and organize the pericentriolar material (PCM), a proteinaceous matrix that anchors microtubule nucleating γ-tubulin complexes (Woodruff et al., 2014). Small electron-dense particles called centriolar satellites, which play a role in centrosomal protein delivery and cellular stress responses (Hori and Toda, 2016), are frequently found in the vicinity of centrioles. Besides their function in the recruitment and organization of the PCM, centrioles are also essential for ciliogenesis. During this process, the older (mother) centriole docks to the cell membrane and extends its peripheral microtubule array, which gives rise to a hair-like cell projection that is referred to as a cilium. In multiciliated cells, cilia formation is initiated from multiple centrioles that have been amplified around electron-dense cellular structures called deuterosomes (Spassky and Meunier, 2017). Cilia have key roles in cellular functions, such as mechanosensing, signal transduction, fluid-flow generation, and cell locomotion (Fliegauf et al., 2007).

Proteomics analyses identified over 100 different proteins associated with human centrosomes (Andersen et al., 2003). Due to a lack of structural information, the exact roles of most of these proteins for the organization and function of the centrosome, as well as their precise mechanism of action, are currently poorly understood. The highly conserved coiled-coil domain-containing protein 61 (CCDC61, also known as variable flagellar number 3, VFL3) is one of these understudied proteins. Unlike wild-type strains, the *vfl3* strain of *Chlamydomonas reinhardtii* does not assemble two cilia per cell, but displays between none and six cilia per cell and consequently shows an altered motility (described as the Vfl^−^ phenotype hereafter) (Wan and Goldstein, 2016, Wright et al., 1983). The *vfl3* mutant has defects in the structure of the basal body complex; it is missing the associated striated fibers and contains altered rootlet microtubules (Wright et al., 1983). Basal body/centriole duplication is also compromised (Marshall et al., 2001). Recent studies on CCDC61 in the unicellular ciliate *Paramecium tetraurelia* showed that the protein plays a crucial role in the orientation of basal bodies and localizes at the interface between basal bodies and ciliary rootlets (Bengueddach et al., 2017). Consistent with these observations, CCDC61 was also shown to be important for the basal body orientation, and the generation of basal feet and ciliary rootlets in the multiciliated ventral epidermis of the flatworm *Schmidtea mediterranea* (Azimzadeh et al., 2012, Basquin et al., 2019), where its absence results in movement defects. Finally, in *Xenopus laevis*, the gene expression of *CCDC61* was found to be upregulated by the expression of Multicilin, which promotes centriole biogenesis in multiciliated cells (Stubbs et al., 2012). These studies point toward a potential role of CCDC61 in the organization of basal bodies in cells with multiple cilia. A recent report suggests that CCDC61 might also be involved in chromatin alignment and mitotic spindle assembly, possibly by anchoring CEP170 (Bärenz et al., 2018, Pizon et al., 2020). However, how CCDC61 functions mechanistically is currently unknown.

Here, we identify CCDC61 as a highly conserved paralog of SAS6, a key organizer of the central scaffold around which centrioles are formed (Leidel et al., 2005). Our crystal structures of CCDC61 demonstrate that it adopts a SAS6-like fold and forms oligomers through two homodimerization domains in a similar way to SAS6: an N-terminal globular head and a parallel coiled-coil domain. However, instead of the spiral/ring assemblies observed with SAS6, CCDC61 assembles into linear filaments with 3-fold, left-handed screw axes *in vitro*. Further analysis of CCDC61 reveals that its coiled-coil domains are capable of directly interacting with microtubules. Residues important for microtubule binding are critical for correct localization of the CCDC61 ortholog VFL3 at basal bodies of *Chlamydomonas* as well as for ciliary function in this organism. Based on these findings, we propose that CCDC61/VFL3 plays a role in scaffolding the assembly of basal body-associated structures throughout eukaryotes.

## Results

### CCDC61 Is a Paralog of SAS6

**Figure 1:**
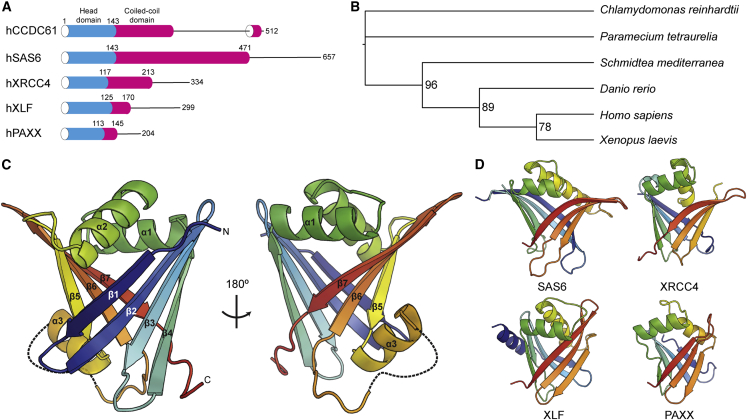
CCDC61 Is an Evolutionally Conserved Protein Paralogous to SAS6 (A) Domain architectures of the XRCC4 superfamily members. Low complexity regions are drawn by lines. (B) A phylogenetic tree of CCDC61 orthologs. Accession numbers of the corresponding amino acid sequences are provided in Table S1. Numbers are bootstrap values. (C) Crystal structure of hCCDC61^1−143^. The structure is presented using a cartoon representation and a rainbow color scheme from the N terminus (N; blue) to the C terminus (C; red). Missing loops are drawn with dotted lines. (D) Crystal structures of the XRCC4 superfamily members SAS6, XRCC4, XLF, and PAXX (PDB: 2Y3W [van Breugel et al., 2011], 1IK9 [Sibanda et al., 2001], 2QM4 [Li et al., 2008], and 3WTD [Ochi et al., 2015], respectively). See also Figures S1, S7 and Table S1.

The XRCC4 protein superfamily is constituted by the centriolar protein SAS6 and the DNA repair proteins XRCC4, XLF, and PAXX. Using a similar computational approach to that used previously to identify PAXX (Ochi et al., 2015), we identified the centrosomal protein CCDC61 (Andersen et al., 2003) as an additional candidate member of this superfamily (Figures 1A and S1A). A phylogenetic analysis of CCDC61 orthologs using PSI-BLAST (Altschul et al., 1997) revealed that CCDC61 is a highly conserved protein present in most Eukaryota that possess centrioles, except for flies and nematodes (Figure 1B; Table S1). Although not present in flies, CCDC61 orthologs are readily identified in other insects that include bees, beetles, and lice (Table S1). Secondary structure analyses of CCDC61 orthologs indicate that they all have an N-terminal domain followed by a discontinuous coiled-coil domain and a low-complexity region, which includes a putative α helix (α9), predicted to be a coiled coil, at the C terminus (Figures 1A and S1B). The sequences of the N-terminal domain and α9 are particularly well conserved across species, whereas those of the coiled-coil and low-complexity region are more variable (Figure S1B).

To gain more insight into the domain organization of CCDC61, we determined the crystal structure of the N-terminal domain of human CCDC61 (hCCDC61^1−143^) at a resolution of 2.6 Å using X-ray crystallography. The structure was solved by the single anomalous dispersion method using seleno-methionine-substituted crystals (Figure 1C; Table 1). As indicated by our bioinformatics analyses, we found that the protein fold of CCDC61 is remarkably similar to the canonical SAS6/XRCC4-like fold, which is characterized by the presence of a seven-stranded β barrel with a helix-turn-helix motif inserted between β4 and 5 (Figures 1C and 1D). CCDC61 has an insertion of an extra α helix (α3) followed by an unstructured loop between β5 and β6 (Figures 1C and S1B), which are unique to CCDC61. We conclude that CCDC61 is a centrosomal protein that constitutes a hitherto unidentified paralog of the XRCC4 superfamily members.

**Table 1:** Data Collection, Phasing and Refinement Statistics of the CCDC61 Crystal Structures

Crystal	hCCDC61^1−143^	zCCDC61^1−168;F129E/D130A^	zCCDC61^1−170^
	SeMet (Peak)	Native	Native
Beamline	DLS I02	MRC LMB	DLS I03
Wavelength (Å)	0.9792	1.5418	0.9762
Resolution (Å)			
Overall	29.68–2.55	44.81–1.97	68.31–2.90
Outer shell	2.66–2.55	2.02–1.97	3.08–2.90
Space group	P22_1_2_1_	P2_1_2_1_2_1_	C222_1_
Unit cell parameters			
a, b, c (Å)	36.877, 68.222, 180.728	55.36, 76.31, 83.24	93.09, 100.56, 135.76
No. of unique reflections	15,498	25,068	14,498
Completeness (%)	99.1 (99.7)^a^	98.2 (96.6)	100 (100)
Redundancy	5.0	7.0	5.9
R_merge_^b^ (%)	7.7 (56.8)	10.2 (86.8)	11.3 (84.9)
<I/σ>	14.7 (2.3)	12.6 (2.4)	9.4 (2.0)
CC½ (%)	99.8 (93.2)	99.7 (75.6)	99.9 (84.0)
Phasing method	Single anomalous diffraction	Molecular replacement	Molecular replacement
FOM	0.339	N/A	N/A
Overall score	39.02	N/A	N/A
Refinement			
PDB:	6HXT	6HXV	6HXY
R_cryst_^c^ (highest shell) (%)	20.81 (35.02)	17.32 (23.49)	19.99 (29.73)
R_free_^d^ (highest shell) (%)	25.60 (41.38)	23.79 (31.77)	25.81 (34.88)
No. of atoms			
Protein atoms	2,900	2,559	2,335
Water molecules	66	299	27
Average B factors (Å^2^)	69.81	34.75	83.16
Ramachandran plot (%)			
Favoured	97.2	97.8	96.6
Outliers	0.0	0.3	0.3
Clashscore	7.59	4.30	8.68
MolProbity overall score	1.72	1.25	1.75
RMSD			
Bond lengths (Å)	0.004	0.008	0.008
Bond angles (°)	1.080	1.098	1.203

FOM, figure of merit; RMSD, root-mean-square deviation.

a The statistics in parentheses are for the highest-resolution shell.

b R_merge_ = Σ_h_|I_h_ − <I>|/Σ_h_I_h_, where I_h_ is the intensity of reflection h, and <I> is the mean intensity of all symmetry-related reflections.

c R_cryst_ = Σ||F_obs_| −|F_calc_||/Σ|F_obs_|, F_obs_ and F_calc_ are observed and calculated structure factor amplitudes.

d R_free_ as for R_cryst_ using a randomly selected 10% for hCCDC61^1−143^ and zCCDC61^1−170^, and 5% for zCCDC61^1−168;F129E/D130A^ of the data excluded from the refinement.

### CCDC61 Forms Higher-Order Assemblies

**Figure 2:**
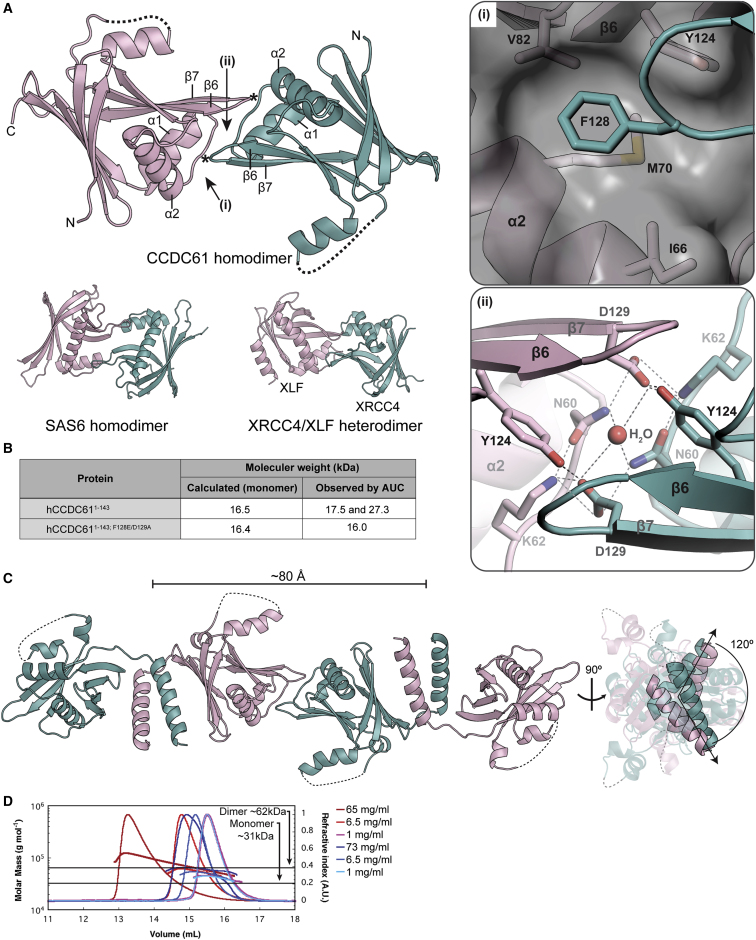
CCDC61 Forms Linear Filaments via Homodimerization Mediated by the Head and Coiled-Coil Domains (A) Crystal structure of the head-to-head homodimer of hCCDC61^1−143^. Missing loops are drawn with dotted lines. Key residues of the interaction interface are indicated by (i) and (ii), of which magnified views are shown in the square boxes on the right. Asterisk indicates the locations of the F128 residues. Dotted lines in panel (ii) indicate hydrogen bonds. Head-to-head dimers of SAS6 and XRCC4/XLF (PDB: 2Y3V [van Breugel et al., 2011] and 3W03 [Wu et al., 2011]) are shown at the bottom. (B) AUC results showing that hCCDC61^1−143^ forms homodimers in solution. (C) Crystal structure of the zCCDC61^1−170^ tetramer. On the right, straight arrows indicate the N-to-C direction of the coiled-coil domains. The angle between the arrows is 120°. (D) CCDC61 forms higher-order oligomers in solution. Size-exclusion chromatography with multi-angle light scattering analysis of His_6_-lipoyl-zCCDC61^1−170^ (red) and His_6_-lipoyl-zCCDC61^1−170; F129E/D130A^ (blue) using a Superdex S200 column at room temperature. Protein concentrations (before injection onto the column) were 1, 6.5, and 65 mg/ml (lightest to darkest red, respectively) and 1, 6.8, and 73 mg/ml (lightest to darkest blue, respectively). The minimum and maximum refractive index values of each chromatography profile were normalized to 0 and 1, respectively. See also Figures S2 and S3.

The asymmetric unit of the hCCDC61^1−143^ crystal contained three copies of protomers that pack tightly against each other through interface regions whose residues are evolutionary conserved (Figure S2A; D1 and D2). Remarkably, one observed CCDC61 homodimer (D1) is highly similar to that formed by the SAS6 head domain (van Breugel et al., 2011, Kitagawa et al., 2011) (Figure 2A). The conserved phenylalanine F128 of hCCDC61 (asterisks in Figures 2A, close up in (i) and S1B, dark blue arrow in the alignment) makes van der Waals interactions with M70 and V82 lining a hydrophobic pocket of the homodimer partner that is constituted by α2, the β hairpin of β5 and 6, and the turns before and after α2 (Figure 2A, in (i)). The dimer interface is further stabilized by an extensive network of salt bridges and hydrogen bonds, including a β zipper formed by residues found between α1 and α2 (Figures 2A and S2B). In this network, the conserved aspartate D129 is central to hydrophilic interactions between two protomers (Figures 2A, in (ii) and S1B, light blue arrow in the alignment).

To test whether homodimer formation of hCCDC61^1−143^ observed *in crystallo* is also observed in solution, we studied the oligomeric state of the protein using analytical ultracentrifugation (AUC). This analysis suggests that hCCDC61^1−143^ exists in a monomer-dimer equilibrium with a *K*_D_ of 170 ± 18 μM (Figures 2B, S2C, and S2D), suggesting a relatively weak binding affinity, similar to that observed with SAS6 (van Breugel et al., 2011, Kitagawa et al., 2011). We next mutated the key residues F128 and D129 of the SAS6-like homodimerization interface of CCDC61 (D1 dimer in Figure S2A) to glutamate and alanine, respectively (hCCDC61^1−143; F128E/D129A^) and subjected the protein to AUC to test whether these residues are important for CCDC61 dimerization. Indeed, dimer formation was abolished in this mutant (Figures 2B and S2C), suggesting that this dimerization interface is dominant in solution, while the other hCCDC61^1−143^ homodimer observed in the asymmetric unit of the crystal (D2 in Figure S2A) appears not to be stable under the experimental conditions.

Due to their overall structural similarity, we wondered whether the head domain of CCDC61 could interact with that of SAS6 to regulate its function. To address this question, we used the recombinant head domains of hCCDC61^1−143^ and the ^15^N-labeled head domain of human SAS6^1−143^ and performed a chemical shift perturbation experiment by nuclear magnetic resonance (NMR) spectroscopy. Our results shown in Figure S2E did not reveal an interaction between the two proteins. Thus, we conclude that the head domain of CCDC61 forms a homodimer but does not heterodimerize with SAS6.

Besides homodimerization of its head domain, SAS6 contains a second dimerization domain constituted by a parallel coiled-coil domain and, through these two interfaces, is able to assemble into a 9-fold symmetric ring structure (van Breugel et al., 2011, van Breugel et al., 2014, Cottee et al., 2015, Kitagawa et al., 2011) except for *C*. *elegans* (Hilbert et al., 2013), where its SAS6 homolog was found *in vitro* to form spiral assemblies instead. To find out whether CCDC61 can form a ring in a similar manner to SAS6, we determined the crystal structure of zebrafish CCDC61 (residues 1–170; zCCDC61^1−170^), which contains both its head and parts of its coiled-coil domain, by X-ray crystallography at a resolution of 2.9 Å (Figure 2C; Table 1). In the crystal, zCCDC61^1−170^ formed a homo-tetramer mediated by the head-to-head and the coiled-coil dimer interactions in an arrangement that would not be compatible with the assembly of a ring. A filament model of zCCDC61^1−170^ suggests that CCDC61 would be able to form protofilaments with a left-handed 3-fold screw axis along the filament, the helical rise of which is ∼80 Å (Figures 2C and S3A). We also obtained a different crystal form of zCCDC61^1−170^ with a hexagonal instead of an orthorhombic lattice. However, due to poor diffraction quality, we could not determine the structure of this crystal form.

To further confirm higher-order oligomer formation of zCCDC61^1−170^ in solution, we subjected this construct, as well as its F129E/D130A mutant that disrupts the head-to-head interaction in zCCDC61, to size-exclusion chromatography with multi-angle light scattering analysis. In this experiment, the His_6_-lipoyl domain tag of each construct was retained to stabilize the corresponding proteins at high concentrations. The results shown in Figure 2D demonstrate that the wild-type, but not the head-to-head dimerization-deficient mutant, was able to form higher-order oligomers beyond the coiled-coil-mediated dimer. Together, these data suggest that CCDC61, like SAS6 (van Breugel et al., 2011, van Breugel et al., 2014, Kitagawa et al., 2011), is able to self-associate into ordered macromolecular assemblies.

Comparison of the structures of zCCDC61, SAS6 (*Leishmania major* [lmSAS6]), and *Caenorhabditis elegans* (ceSAS6) and human XRCC4/XLF by superposition of their head domains showed that the difference between the exact higher-order assemblies formed by these proteins originates from (1) altered relative orientation angles between their head domains and (2) altered relative orientation angles between the head and coiled-coil domains (Figures S3A and S3B). When defining as z axis (z) the rotation axis required to bring the second head domains of zCCDC61 and lmSAS6 into superposition (Figure S3B, top panel), the corresponding rotation axis between the head domains of zCCDC61 and ceSAS6 also corresponds to z, whereas that between zCCDC61 and hXRCC4/XLF is about 10° off relative to z (Figure S3B, top and lower left panels). In comparison with the relative angle between the head and coiled-coil domain of lmSAS6, the corresponding angle of the other XRCC4 superfamily members is also altered: The coiled-coil domain orientation of zCCDC61 and hXRCC4 deviate in the opposite direction to those of ceSAS6 and hXLF (Figure S3B, lower right panel). Previous observations of structures of SAS6 suggest that the relative orientation angle between the head and coiled-coil domains in particular determines the symmetry of the resulting filaments (Hilbert et al., 2013). Since both head-to-head and head-to-coiled-coil orientation angles appear to be able to change independently from each other, a confirmation of this hypothesis will require further structural information on the superfamily members. Nevertheless, we conclude that two separate dimerization domains of the XRCC4 superfamily proteins allow them to form filaments with different symmetries and helical parameters through mutations of residues involved in the head-to-head and head-to-coiled-coil interactions.

### The Coiled-Coil Domain of CCDC61 Binds to Microtubules

**Figure 3:**
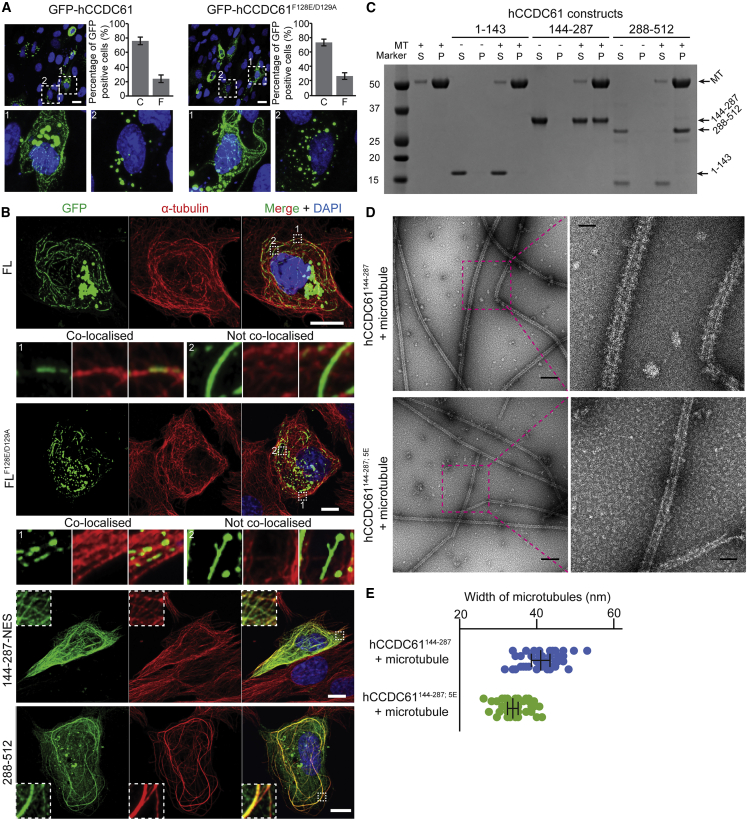
CCDC61 Binds Microtubules (A) Fluorescent images of RPE-1 cells, transiently overexpressing GFP-hCCDC61 or hCCDC61^F128E/D129A^, showing the different CCDC61 localization patterns observed under these conditions. Bar graphs show the percentage of GFP-positive cells containing clusters-only “C” versus filament-containing cells “F” (n = 279 for GFP-hCCDC61 and n = 468 for GFP-hCCDC61^F128E/D129A^ counted from three biological replicates). Error bars are standard deviations. Positions of blow-up images labeled with 1 (filament-containing cell) and 2 (cluster-only cell) are indicated with white-dotted squares in the top panels. Scale bars, 20 μm. (B) Transiently overexpressed hCCDC61 colocalizes with microtubules in cells. Immunofluorescent images of RPE-1 cells transiently overexpressing GFP-hCCDC61, GFP-hCCDC61^F128E/D129A^, GFP-hCCDC61^144−287−NES^, and GFP-hCCDC61^288−512^. Anti-GFP staining is shown in green, microtubule staining in red. Magnified views of the regions indicated by the white-dotted squares in the merged images are shown either below (GFP-hCCDC61 and GFP-hCCDC61^F128E/D129A^) or as insets (GFP-hCCDC61^144−287−NES^ and GFP-hCCDC61^288−512^). Displayed are representative images acquired from a total of 14, 8, 10, and 11 different RPE-1 cells for GFP-hCCDC61, GFP-hCCDC61^F128E/D129A^, GFP-hCCDC61^144−287−NES^, and GFP-hCCDC61^288−512^, respectively. Scale bars, 10 μm. (C) Coiled-coil and C-terminal regions of hCCDC61 bind microtubules *in vitro*. Coomassie-stained SDS-PAGE gel showing a co-pelleting assay of taxol-stabilized microtubules with the head domain (1–143), PAXX-fused coiled-coil domain (144–287), or the C-terminal region (288–512) of hCCDC61. S and P indicate supernatant and pellet fraction, respectively. (D) The coiled-coil domain of hCCDC61 directly binds microtubules. Negative-stain EM micrographs of microtubules that show their decoration with a layer of PAXX-hCCDC61^144−287^ that is not observed with the corresponding 5E mutant of CCDC61. Scale bars, 200 and 50 nm in the overview panels (left) and the magnified panels (right), respectively. (E) Quantification of the widths of microtubules decorated by PAXX-hCCDC61^144−287^ or in the presence of PAXX-hCCDC61^144−287; 5E^ from (D). Widths of five different positions of ten microtubules were measured for each construct. Each point (blue for PAXX-hCCDC61^144−287^ and green for PAXX-hCCDC61^144−287; 5E^) represents a measured width at each position. Error bars (standard deviations from the mean) are shown in black lines with flat arrow ends. See also Figure S4.

Next, we overexpressed GFP-hCCDC61 in human RPE-1 cells and performed a fluorescence-imaging experiment to better understand the behavior of full-length CCDC61. The majority of the protein-formed clusters and ∼25% of GFP-hCCDC61-expressing cells showed filament-like structures in the cytoplasmic region (Figure 3A), although the extent and type of cluster formation by GFP-hCCDC61 varied widely among cells perhaps due to differences in expression levels. We hypothesized that the observed filament formation *in vivo* might be mediated by head-to-head dimer formation of CCDC61 as in our crystal structure (Figure 2A). However, when we disabled head-to-head dimer formation using the GFP-hCCDC61^F128E/D129A^ mutant, we still observed a similar variety of localization patterns compared with the wild-type experiment (Figure 3A). Some of the hCCDC61 filaments formed *in vivo* upon overexpression were reminiscent of cytoplasmic microtubules. Immunofluorescence experiments against GFP-hCCDC61 and microtubules in RPE-1 cells indeed showed colocalization between most, but not all, of the observed GFP-hCCDC61 filaments and microtubules (Figures 3B and S4A). Similar observations were made when we overexpressed the GFP-hCCDC61^F128E/D129A^ mutant (Figures 3B and S4A).

The relative proportion of GFP-hCCDC61 filament- or cluster-forming cells was largely unchanged when the microtubule-destabilizing agent nocodazole or the microtubule-stabilizing agent taxol (Figure S4B) were added to cells, indicating that CCDC61 bound microtubules might be protected against the action of microtubule poisons and that the exchange rate between the different CCDC61 pools might be low. In agreement, live cell imaging of RPE-1 cells showed that GFP-hCCDC61 filaments persisted in the presence of 5 μM nocodazole over the course of 3 h (Figure S4C).

We speculated that the coiled-coil and/or the C-terminal regions of hCCDC61 are responsible for its microtubule association. To test this hypothesis, we overexpressed either the coiled-coil domain (144–287) or the C-terminal region (288–512) of hCCDC61 as GFP-tagged constructs in RPE-1 cells and carried out immunofluorescence experiments. The coiled-coil domain construct was fused to an NES to avoid its mis-localization to the nucleus. Our results indeed showed that both the coiled-coil and C-terminal regions of CCDC61 colocalize with microtubules (Figures 3B and S4A).

Since microtubule association in cells might indicate microtubule binding by CCDC61, we sought to perform a direct binding assay to address this question. To this end, we purified the hCCDC61 head domain as well as its coiled-coil and its C-terminal region as recombinant proteins and performed microtubule co-pelleting assays using taxol-stabilized microtubules *in vitro*. To stabilize the coiled-coil domain of hCCDC61, we fused it to the C terminus of the DNA repair protein PAXX (residues 1–137) whose head domain is structurally similar to that of CCDC61 but does not dimerize (Ochi et al., 2015). Our results suggest that both coiled-coil and C-terminal domain of CCDC61 are indeed able to directly bind to microtubules *in vitro* (P (pellet) in Figure 3C), whereas the head domain is unable to do so and remains in the supernatant fraction (S (supernatant) in Figure 3C).

Positively charged residues of microtubule-associated proteins frequently play a role in microtubule binding (Cooper and Wordeman, 2009). Intriguingly, the coiled-coil domain of hCCDC61 (residue 144–287; α4–7) has an overall positive charge (theoretical pI ∼10.5). To identify residues involved in microtubule binding by CCDC61, we mutated five conserved positively charged residues in α7 (K259, R263, R266, R268, and K270, Figure S1B, red arrows) and repeated the microtubule co-pelleting assay. Mutation of these residues largely abolished the microtubule binding activity of hCCDC61 (Figure S4D) without affecting the overall structure of CCDC61 or its general ability to form oligomers (Figures S4E and S4F), despite some destabilization of the mutated coiled-coil domain on its own compared with the corresponding wild-type construct *in vitro* (Figure S4F). The positively charged residues in the coiled-coil domain of hCCDC61 might interact with the negatively charged residues of the tubulin C termini. To test this, we removed the C-terminal tails of tubulin from taxol-stabilized microtubules using the protease subtilisin (Serrano et al., 1984) (Figure S4G) and repeated the microtubule co-pelleting assay. The result showed that the coiled-coil domain of CCDC61 indeed mainly interacts with the tubulin C termini (Figure S4H). Intriguingly, upon overexpression of the 5E mutant of GFP-hCCDC61 in RPE-1 cells, we did not observe CCDC61 filament formation (Figure S4I), while CCDC61 clusters were still observed. This indicates that *in vivo* and in the full-length context, the α4–7 part of CCDC61 comprises the dominant microtubule binding activity in CCDC61 and that filament formation of CCDC61 is largely mediated by its microtubule binding.

We also mixed the PAXX-stabilized α4–7 coiled-coil domain of hCCDC61 with taxol-stabilized microtubules and subjected the mixture to electron microscopic analysis using negative staining. The micrograph shown in Figure 3D demonstrates that microtubules were decorated with hCCDC61 while this decoration was not observed when we used the equivalent construct carrying the 5E mutation (average widths of microtubules: 40.98 ± 2.55 nm [mean ± standard deviation] and 33.97 ± 1.32 nm, respectively [Figure 3E]). We note that the average width of microtubules in the presence of the 5E mutant seems to be larger than the canonical diameter of microtubules (24 nm). However, this might be due to the negative staining of microtubules on carbon-coated electron microscopic grids as similar, wider microtubule widths also have been observed by others (Reid et al., 2017, Shibata et al., 2012). Together, these results suggest that CCDC61 primarily binds to microtubules by engaging the C-terminal tails of tubulin via conserved positively charged residues of α7 of CCDC61.

### CCDC61 Localizes to Basal Bodies

**Figure 4:**
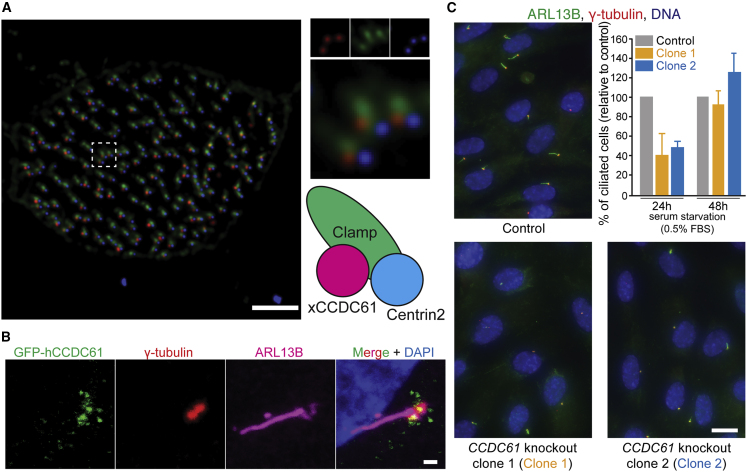
CCDC61 Associates with Basal Bodies and Plays a Role in Ciliogenesis (A) xCCDC61 associates with basal bodies and rootlets in multi-ciliated epidermal cells of *Xenopus* embryos. A fluorescent image of a *Xenopus* embryo expressing xCCDC61-RFP (red), the basal body component Centrin2-BFP (blue), and the rootlet component Clamp-GFP (green). Scale bar, 3 μm. (B) Location of hCCDC61 at the periphery of basal bodies of primary cilia. Immunofluorescent image of an RPE-1 cell transiently overexpressing GFP-hCCDC61. Co-immunofluorescent staining was performed against GFP (green), basal bodies (γ-tubulin, red), and the ciliary axoneme (ARL13B, magenta). Scale bar, 1 μm. (C) Ciliated cells of control and *CCDC61*-knockout RPE-1 cells. Immunofluorescent images show representative immunofluorescent images used for quantifications of ciliogenesis of primary cilia. Scale bar, 10 μm. The bar graph shows that ciliogenesis was delayed in the *CCDC61* knockout cells. Data shown correspond to three biological replicates (total cell counts n = 1,181, 1,103, and 1,008 for control, clone 1 and clone 2 cells after 24-h serum starvation respectively, and n = 1,151, 1,046 and 1,242 for control, clone 1 and clone 2 after 48-h serum starvation, respectively). Percentages are relative to control cells. Bar graphs show mean ± standard deviation. See also Figure S5.

In the ciliate protist *Paramecium tetraurelia*, previous studies have demonstrated that CCDC61 localizes to basal body-associated substructures, such as rootlets or striated fibers (Bengueddach et al., 2017). To find out whether hCCCD61 would also be found associated with basal bodies in multicellular organisms, we checked the distribution of CCDC61 in multiciliated epithelial cells of *Xenopus laevis* embryos expressing *Xenopus* CCDC61 (xCCDC61) fused to the N terminus of RFP. Three-color imaging of xCCDC61-RFP, Centrin2-BFP (marking the distal centriole region) and Clamp-GFP (marking the rootlet) in these multiciliated frog embryos demonstrated that, like its unicellular orthologs, xCCDC61 associates with the proximal part of basal bodies in a polarized manner that is close to, but distinct from rootlets (Figure 4A). We also performed immunofluorescent imaging of GFP-hCCDC61 overexpressed in RPE-1 cells under serum-starved conditions in which these cells form a single, non-motile primary cilium. This analysis showed that hCCDC61 can also localize to the periphery of human basal bodies (Figure 4B). To investigate the function of hCCDC61 in this cell line, we generated hCCDC61-deficient RPE-1 cells using CRISPR/Cas9 (Figure S5A). Although we did not observe obvious defects in proliferation, cell-cycle progression and centrosome or centriole numbers (Figures S5B–S5D), we observed a delay in the formation of primary cilia both in these hCCDC61-deficient RPE-1 cells (Figure 4C) and also in RPE-1 cells depleted of hCCDC61 by RNA interference (Figure S5E). The delay in cilia formation that is observed in hCCDC61-deficient cells could potentially impact developmental processes. However, normal-looking cilia assemble eventually in RPE-1 cells, which suggests that hCCDC61 plays a role in ciliogenesis but is not essential for the generation of primary cilia. Together, these results suggest that the localization of CCDC61 to basal bodies is evolutionarily conserved.

### Characterization of *Chlamydomonas* Strains Carrying Mutations in the CCDC61 Ortholog VFL3

CCDC61 orthologs play an important role in the functioning of motile cilia in different model organisms (Azimzadeh et al., 2012, Bengueddach et al., 2017, Wright et al., 1983). We wondered whether the filament-forming/microtubule binding activity of CCDC61 would be functionally important in this respect. To answer this question, we used *Chlamydomonas* as a model organism because strains containing defective *VFL3* (its *CCDC61* ortholog) are available and the mutant phenotypes have been well characterized (Hoops et al., 1984, Keller et al., 2010, Marshall et al., 2001, Wright et al., 1983). Furthermore, VFL3 shares 36% sequence identity with hCCDC61 and key residues involved in head-to-head interaction and microtubule binding are conserved (Figure S1B, blue and red arrows). Through Sanger sequencing, we identified a nonsense mutation (AAG to TAG) in the *VFL3* gene in the original mutant, which we named *vfl3-1*. The nonsense mutation (K497X; Figure S1B, green arrow) is found in exon 8. We also obtained an insertion mutant, LMJ.RY0402.091002, which has an insertion of an exogenous DNA cassette that confers paromomycin resistance, in intron 7 of *VFL3*, from the *Chlamydomonas* CLiP mutant library (Li et al., 2016). In 20 tetrads of this insertional mutant crossed to wild-type, we observed complete co-segregation of the paromomycin resistance phenotype and the Vfl^−^ phenotype. Therefore, we considered this insertion mutant a second allele of *vfl3* and renamed it *vfl3-2*.

**Figure 5:**
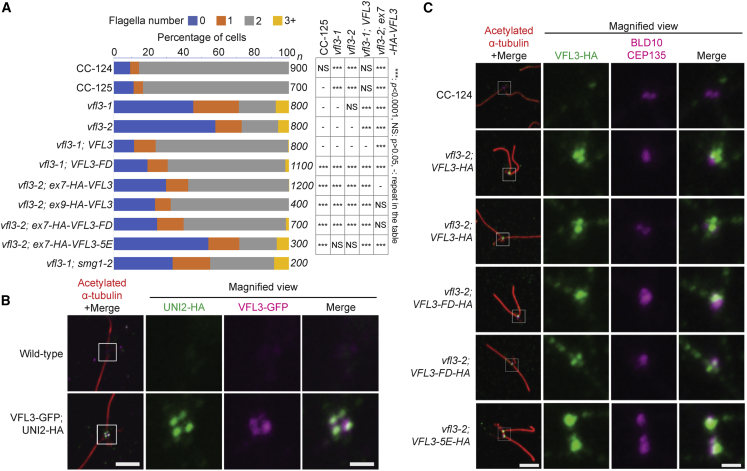
*Chlamydomonas* VFL3 Protein Localizes to Basal Bodies and the Proximal Ends of Flagella (A) Rescue of abnormal flagella numbers in *vfl3* strains by wild-type *VFL3*. Bar chart showing flagella numbers observed in wild-type strains (CC-124 and CC125), mutant strains (*vfl3-1* and *vfl3-2*), and the *vfl3-1* and *vfl3-2* strains expressing VFL3 constructs in *Chlamydomonas*. The numbers of cells “n” used for calculating ratio flagella numbers are shown on the right side of the chart. A χ^2^ test was used to determine if the number of cells with zero flagella was significantly different. NS, not significant; ^∗∗∗^p < 0.0001. (B) Wild-type VFL3 protein localizes to *Chlamydomonas* basal bodies. In the first column, cells were stained with acetylated α-tubulin (red) for cilia and rootlet microtubules, anti-HA (green) for UNI2, and anti-GFP (magenta) for VFL3. Scale bar, 4 μm. Magnified views (4×) of the basal body regions (white boxes) are shown on the other three columns. Scale bars, 1 μm. (C) Localization of VFL3 is affected in the 5E mutant. In the first column, cells were stained with acetylated α-tubulin (red) for cilia and rootlet microtubules, anti-HA (green) for wild-type and mutant VFL3, and anti-BLD10/CEP135 (magenta). Scale bar, 4 μm. Magnified views (4×) of the basal body regions (white boxes) are shown on the other three columns. Scale bar, 1 μm. See also Figure S6.

In both mutant alleles, we quantitated the Vfl^−^ phenotype. We observed that in an asynchronous culture that 7% and 6% of *vfl3-1* and *-2*, respectively, had more than two flagella. This is a phenotype not seen in any wild-type strains. Moreover, the two mutants had an increased number of cells with no flagella (45% and 58% compared with wild-type with 10%) or one flagellum (26% and 15% versus 5% for wild-type) (Figure 5A). By immunofluorescence experiments (Figure S6A), the wild-type (CC-124) exhibited normal distal striated fibers (stained with antibodies to centrin, which is found in the distal striated fibers in *Chlamydomonas* [Dutcher and O'Toole, 2016]) and biciliated cells (stained by acetylated α-tubulin), whereas we noted abnormal striated fibers and abnormal cilia number in the *vfl3-2* mutant strain as reported previously in *vfl3-1* (Wright et al., 1983).

Analysis of the *VFL3* transcripts in *vfl3-1* revealed that this mutant contains a full-length transcript (Figure S6B). In *vfl3-2*, the *VFL3* mRNA is truncated and contains only exons 1–7, which are located upstream of the insertional cassette (Figure S6B). We found that transformation of the wild-type *VFL3* gene into *vfl3-1* restores normal ciliary numbers (Figures 5A; Table 2). To detect the VFL3 protein, a 3xHA epitope tag was introduced within either exon 7 (ex7-HA) or exon 9 (ex9-HA) of *VFL3* and both tagged *VFL3* transgenes were integrated into the *vfl3-2* strain, where they gave rise to full-length *VFL3* transcripts (Figure S6B). However, the tagged genes only partially rescued the mutant phenotype (Figure 5A). The number of cells with zero flagella is not restored to wild-type levels (p < 0.0001) by a χ^2^ test (Figure 5A). The transgene did restore the striated fiber phenotype in *vfl3-2* (Figure S6A). Therefore, we confirmed that the phenotypes of the *vfl3* strains are due to the *VFL3* gene defects.

**Table 2:** Summary of *Chlamydomonas* Transformation with Various Constructs

Strain	Construct	No. of APHVIII Transformants^a^	No. of *vfl3* Rescued Strains
*vfl3-1*	VFL3	875	3
	VFL3-FD	316	1 (partial rescued)
	VFL3-5E	449	0
*vfl3-2*	VFL3	666	8
	VFL3-FD	446	1 (partial rescued)
	VFL3-5E	970	0
	Ex7-HA-VFL3	658	5
	Ex9-HA-VFL3	133	1
	Ex7-HA-VFL3-FD	227	1 (partial rescued)
	Ex7-HA-VFL3-5E	221	0
Total		4961	19

a Transformants are identified by co-transformation with the *APHVIII* gene that confers resistance to the antibiotic paromomycin.

### The Basic Amino Acids Involved in Microtubule Binding Are Important for VFL3 Function and Localization

Immunoblots of *Chlamydomonas* whole-cell extracts from various transformants that carry either ex7-HA- or ex9-HA-tagged VFL3 with an anti-HA antibody reveal a single polypeptide with the expected size of ∼85 kDa (Figures S6C and S6D), which is absent in extracts of wild-type cells (CC-124) (Figure S6C). Immunofluorescence of NFAPs (nucleoflagellar apparatus) (Wright et al., 1985) in multiple, independent transformants shows that VFL3 (Figures 5B, magenta and 5C, green) localizes to both the basal bodies (Figures 5B, green and 5C, magenta). To determine the precise location of VFL3 in the basal bodies, we co-stained VFL3 with UNI2, a protein that localizes to the distal end of the basal bodies (Figures 5B; Video S1) (Piasecki and Silflow, 2009); and BLD10/CEP135, which localizes to the cartwheel of the basal bodies at their proximal end (Matsuura et al., 2004) (Figure 5C). Our results suggest that VFL3 does not overlap completely with either UNI2 or BLD10 and is likely distributed along the full length of the basal bodies.

Video S1. Localization of Wild-Type VFL3 to *Chlamydomonas* Basal Bodies, Related to Figure 5 Cells were stained with acetylated α-tubulin (red) for cilia and rootlet microtubules, anti-HA (green) for UNI2, and anti-GFP (magenta) for VFL3. z stacked images start from the distal ends of basal bodies (labeled by positions of UNI2) and move toward the proximal ends. Scale bar, 0.8 μm.

We next asked whether filament formation and microtubule binding of VFL3 are important for its function and localization. We first generated a strain containing the F126E and D127A mutations in VFL3 (VFL3-FD), which are equivalent to the F128E/D129A mutation in hCCDC61 that disrupts its head-to-head homodimerization. We transformed the *VFL3-FD* transgene (untagged or HA tagged [Figure S6D]) into both *vfl3-1* and *vfl3-2* cells, and observed a partial rescue of the mutant phenotype in the *vfl3-1* and *vfl3-2* cells, respectively (Figure 5A). The untagged *VFL3* transgene, and the untagged FD mutant transgene are significantly different from the mutant parent, while the untagged FD mutant is also significantly different from the strain with the wild-type transgene (p < 0.0001) based on the number of cells with zero flagella (Figure 5A) by a χ^2^ test. The HA-tagged FD mutant was not significantly different from the HA-tagged wild-type transgene in the *vfl3-2* strain. The difference between tagged and untagged FD mutants might be due to the HA tag partially interfering with the protein function. About 2% of cells contain more than two cilia compared with less than 0.1% in wild-type cells. These data suggest that there is a partial rescue of the Vfl^−^ phenotype by the *VFL3-FD* transgene. The VFL3-FD-HA protein localizes to the basal body region (Figure 5C), similar to what we observed in wild-type VFL3-HA. We also mutated the five basic amino acids residues (K266, R270, K273, R275, and R277), whose equivalents in hCCDC61 are involved in microtubule binding, to glutamates (VFL3-5E). Co-transformation of the *VFL3-5E* transgene into the *vfl3-1* and *vfl3-2* strains with the *APHVIII* gene, which confers resistance to the antibiotic paromomycin, failed to yield any strains with a rescued phenotype in ∼1,400 drug-resistant transformants (Table 2). To investigate whether the failure to rescue is caused by an absence of expression of the transgene or by the 5E mutation, we co-transformed an HA-tagged version of *VFL3-5E* into *vfl3-2* and screened ∼200 drug-resistant transformants by immunoblot and immunofluorescence (Figures 5C and S6D). We obtained a single transformant that showed expression of the HA-VFL3-5E protein but the 5E mutant strain was not significantly different from the mutant *vfl3-2* parent (p = 0.68) based on the number of cells with zero flagella (Figure 5A) by a χ^2^ test (Figure 5A). We observed accumulation of HA-VFL3-5E around the basal body region with BLD10/CEP135 (Figure 5C). Thus, our results suggest that the microtubule binding region of VFL3/CCDC61 plays a critical role in its function and accurate localization in living cells.

## Discussion

**Figure 6:**
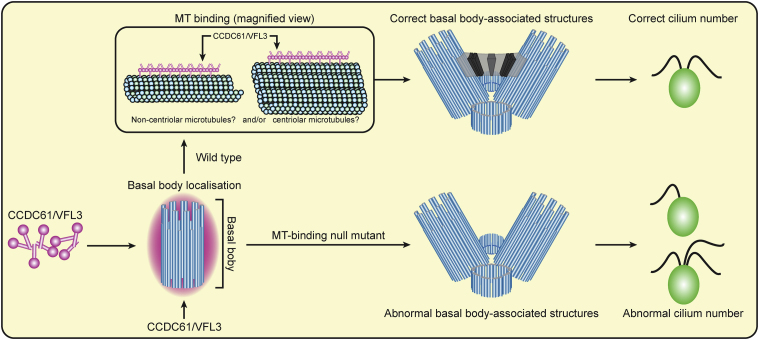
Model of the Role of CCDC61 in Ciliary Function (in *Chlamydomonas*) CCDC61 localizes to the basal body and forms filaments that bind to centriolar and/or non-centriolar microtubules. This facilitates striated fiber formation and the correct formation of basal body-associated structures, and therefore, results in the correct cilium number. A CCDC61 mutant that does not bind microtubules (MT-binding null mutant) still localizes to the basal body region. However, the mutant is incapable of facilitating striated fiber formation, leads to incorrect formation of basal body-associated structures, and therefore causes abnormal cilium numbers.

Here we provide a detailed structural and biochemical characterization as well as a functional analysis of the centrosomal protein CCDC61. CCDC61 is a paralog of the centriolar protein SAS6, forms higher-order oligomers and is capable of binding microtubules *in vitro* and *in vivo*. Furthermore, we demonstrate that the CCDC61 ortholog in *Chlamydomonas*, VFL3, localizes at basal bodies. Our functional studies in cells suggest that microtubule binding of VFL3/CCDC61 is important for its correct localization to basal bodies and its function *in vivo*, whereas its head-to-head interaction appears not to be critical but plays a role in ensuring faithful formation of basal bodies *in vivo*. Collectively, our data suggest that the main function of CCDC61 might lie in the organization of basal body-associated structures (Figure 6).

Our biochemical and structural data unambiguously demonstrate that CCDC61 belongs to the XRCC4 superfamily of proteins. Members of this superfamily have a centrosomal/centriolar function (SAS6 [Leidel et al., 2005] and CCDC61 [Andersen et al., 2003, Wright et al., 1983]) or play crucial roles in the NHEJ DNA repair pathway (XRCC4 [Li et al., 1995], XLF [Ahnesorg et al., 2006, Buck et al., 2006] and PAXX [Craxton et al., 2015, Ochi et al., 2015, Xing et al., 2015]). Their protein architecture consists of an N-terminal head domain followed by a coiled-coil and C-terminal low-complexity region. A structure-guided sequence alignment of the head domains of the human XRCC4 superfamily members showed that the sequence identities between them are below 20% (Figures S7A and S7B). However, they share a conserved sequence motif (Figure S7A, red-dotted rectangle), which has previously been named the PISA motif (Leidel et al., 2005). The motif is likely to be critical for the functions of the XRCC4 superfamily members because homozygous mutations in this motif in *XRCC4*, *XLF*, or *SAS6* cause growth defects (Buck et al., 2006, Khan et al., 2014, Murray et al., 2015). Their similarity also extends to the ability of the superfamily members (except for PAXX), to form protofilaments using two dimerization interfaces provided by the head and the coiled-coil domains. Head-to-head dimerization in all these cases occurs with a low binding affinity, suggesting that these proteins need to be enriched locally and/or be stabilized by other molecules to efficiently form faithful higher-order assemblies. In agreement with this, the protein concentration of CCDC61 in HeLa cells appears to be very low (Bauer et al., 2016). SAS6 assembly, for instance, is probably aided by its interaction with CEP135 and STIL (Dzhindzhev et al., 2014, Lin et al., 2013, Ohta et al., 2014), whereas for the XRCC4/XLF complex this function is exerted by its associations with DNA ligase IV, Ku70/80 and DNA (Ochi et al., 2014). Putative CCDC61 binding proteins, such as CEP170 (Bärenz et al., 2018, Pizon et al., 2020) might play an equivalent role in CCDC61. Thus, the overarching principles of higher-order oligomerization and stabilization by other proteins appears to be conserved among the XRCC4 superfamily members. It is worth mentioning that the head domain of hCCDC61 contains a conserved surface area that is not directly involved in D1 dimer formation as described in Figures 2A and S2A. Instead, it contributes to the formation of the D2 dimer that we observed in the asymmetric unit of the hCCDC61^1−143^ crystal, but not in solution (Figures 2B, S2A, and S2C). This surface might be involved in a protein-protein interaction between CCDC61 and another protein. Alternatively, it is possible that the D2 dimer exists *in vivo* under high local concentrations and that it might facilitate formation of CCDC61 filament bundles. Indeed, when we superposed two CCDC61 filaments onto the D2 structure, we found that these filaments do not clash strongly with each other (Figure S7C).

Our phylogenetic analysis demonstrates the presence of CCDC61 in most ciliated eukaryotes, except for flies and nematodes. Interestingly, the conservation pattern of CCDC61 (Table S1) is very similar to those of δ- and ε-tubulins (Hodges et al., 2010), which are important for centriolar doublet and triplet microtubule formation (Dutcher et al., 2002, Dutcher and Trabuco, 1998, Wang et al., 2017). However, our data argue against a central role of CCDC61 in centriole/centrosome duplication in human cells (Figures S5B–S5D). These results agree with findings in the flatworm *S. mediterranea* (Azimzadeh et al., 2012), but contrast with reports from the unicellular *Chlamydomonas* (Marshall et al., 2001) and *Paramecium* (Bengueddach et al., 2017). The difference between these studies possibly stems from the fact that centrioles are duplicated as basal bodies in these organisms, whereas they are duplicated through the centrosomal or deuterosomal pathway in humans and planaria. Thus, CCDC61 itself might not be part of the core centriole duplication machinery in these unicellular organisms, but rather be important for the maintenance of the basal body-associated architectures that are needed for the faithful recruitment of this duplication machinery to probasal bodies.

Consistent with a role of CCDC61 in basal body function, we observed localization of CCDC61 at basal bodies of primary cilia of human RPE-1 cells, motile cilia in green algae, and motile cilia of multiciliated cells of frog embryos (Figures 4A, 4B, 5B, and 5C). Our data, as well as evidence from other systems in which CCDC61 has been studied, suggest that the main function of CCDC61 is related to basal body function in cells with motile cilia. In the motile ciliate *Paramecium*, CCDC61 localizes at the interface between basal bodies and striated rootlets and is important for their organization (Bengueddach et al., 2017). These basal body-associated structures play a role in basal body positioning at the cell cortex and in probasal body assembly (Hoops et al., 1984). A specific role of CCDC61 in the anchoring of basal bodies in multiciliated cells is also suggested by experiments in the planarian *S. mediterranea*. Planaria move by gliding on a ventral array of multiciliated cells (Azimzadeh and Basquin, 2016). Knockdown of CCDC61 in *S. mediterranea* was found to result in an abnormal direction of locomotion (Azimzadeh et al., 2012) due to basal body mis-orientations caused by a failure to generate basal feet and ciliary rootlets correctly (Basquin et al., 2019).

This notion is in agreement with previous studies on the *Chlamydomonas vfl3-1* strain that suggest that VFL3 is crucial for the faithful organization of proximal and distal striated fibers as well as rootlet microtubules (Hoops et al., 1984, Wright et al., 1983). We also confirmed this using the insertional mutant strain (*vfl3-2*). Interestingly, the *vfl3-1* strain carries a premature stop codon after K497, which would retain the head and coiled-coil domains of VFL3 but not α9. Similarly, our transcript analysis suggests that the *vfl3-2* strain may carry a truncated protein that retains the first 406 amino acids of VFL3, 22 amino acids downstream of the α8 helix. Thus, this could be indicative of a crucial role of α9 in VFL3 function, given the mutant phenotype in both strains. However, since we could not obtain reliable antibodies that detect VFL3, we cannot exclude the possibility that the truncated VFL3 gene product is destabilized in these strains explaining the observed phenotype.

Although our crystal structures of CCDC61 fragments *in vitro* suggest that CCDC61 forms filaments (Figure 2C), we could not visualize these *in vivo*. However, a VFL3/CCDC61 allele with a disabled head-to-head dimerization (VFL3-FD) was unable to completely rescue the Vfl^−^ phenotype (Figure 5A) in *Chlamydomonas*, arguing for a functional role of this interface. Furthermore, since both *vfl3-1* and *vfl3-2* strains retain intact exons 1–7 of VFL3 (residue range 1–406), a heterodimer between the putative truncated VFL3 and the rescue construct might form and be partially functional, potentially accounting for the weak phenotype observed in the VFL3-FD strain. Intriguingly, in the filaments formed by CCDC61 *in vitro*, the distance between the projecting coiled-coil domains of CCDC61 that point in the same direction is about 24 nm, which corresponds to three times the 8-nm repeat of tubulin dimers in microtubules (Figure 2C). This periodicity might facilitate microtubule interaction by the CCDC61 coiled-coil domain.

Despite the weak phenotype observed in the VFL3-FD strain, we speculate that the ability of CCDC61/VFL3 to form higher-order assemblies might aid scaffold formation of the protein through which basal body-associated substructures are anchored or helped to stay in place under the mechanical stresses acting on motile cilia (Figure 6). CCDC61/VFL3 might interact with a centrosomal and basal body-specific protein that is yet to be identified, and scaffold the protein with microtubules to construct regularly aligned basal body-associated structures. Two groups recently proposed that CCDC61 interacts with CEP170 and might play a role in the subdistal appendage function of centrioles (Bärenz et al., 2018, Pizon et al., 2020). While our manuscript was under review, Pizon and colleagues also reported CCDC61 association with microtubules (Pizon et al., 2020), in agreement with our data.

Our study also raises several questions, particularly, whether, *in vivo*, CCDC61 forms protofilaments as observed *in crystallo* and, if so, what their exact role is. How does microtubule binding of CCDC61 assist in the assembly and organization of basal body-associated structures? Further research efforts are required to elucidate the exact function and the molecular mechanisms of CCDC61 that underlie the biogenesis of these structures.

## STAR★Methods

### Key Resource Table

**Table:** 

REAGENT or RESOURCE	Source	Identifier
**Antibodies**		

Rabbit anti-ARL13B	Proteintech	17711-1-AP
Mouse anti-alpha-tubulin	Sigma-Aldrich	T9026
Rabbit anti-acetylated-alpha-tubulin	Abcam	ab179484
Mouse anti-gamma-tubulin	Sigma-Aldrich	T6557
Chicken anti-GFP	Abcam	ab13970
Mouse anti-GFP	Thermo Fisher Scientific	A11120
Rabbit anti-HA	Gift from Dr Manu Hedge	N/A
Rat anti-HA	Roche	118674230001
Mouse anti-centrin	Gift from Dr Jeffrey L. Salisbury	N/A
Mouse anti-centrin 3	Abnova	H00001070-M01

**Bacterial Strains**		

BL21(DE3)	New England Biolabs	C2527
C41(DE3)	Miroux and Walker, 1996	N/A
Rosetta (DE3)	Gift from Dr John Kilmartin	N/A

**Chemicals, Peptides, and Recombinant Proteins**		

D-MEM Glutamax	Thermo Fisher Scientific	Catalog # 10566016
D-MEM/F-12, supplied, GlutaMAX, sodium carbonate	Thermo Fisher Scientific	Catalog # 31331028
D-MEM/F-12 without phenol red	Thermo Fisher Scientific	Catalog # 21041025
Opti-MEM	Thermo Fisher Scientific	Catalog # 31985062
CloneAmp HiFi Premix	Clontech	Catalog # 639298
In-Fusion HD cloning	Clontech	Catalog # 638933
RNeasy Mini Kit	Qiagen	Catalog # 74104
RNase-free DNase I	Thermo Fisher Scientific	Catalog # EN0521
SuperScript IV VILO Master Mix	Thermo Fisher Scientific	Catalog # 11756050
QuickExtract DNA extract solution	Cambio	Catalog # QE0950
0.1% poly-L-Lysine	Sigma-Aldrich	Catalog # P8920
Ni-NTA resin	Expedeon	Catalog # ANN0100
Ni-NTA	Qiagen	Catalog # 30210
Glutathione sepharose 4B	GE Healthcare Life Sciences	Catalog # 17075601
NHS-activated sepharose 4 Fast Flow	GE Healthcare Life Sciences	Catalog # 17090601
Tev protease	Homemade	N/A
GST-PreScission protease	Homemade	N/A
Tubulin	Gift from Dr Andrew Carter	N/A
Subtilisin A	Sigma-Aldrich	Catalog # P5380
Monastrol	Sigma-Aldrich	Catalog # M8515
ProLong Diamond Antifade Mountant	Thermo Fisher Scientific	Catalog # P36970
Fluoromount-G	Southern Biotech	Catalog # 0100-01
Hoechst 33342	EMP Biotech	Catalog # F-0409
PEI	Polysciences	Catalog # 24765
Lipofectamine 3000	Thermo Fisher Scientific	Catalog # L3000001
Lipofectamine RNAiMAX	Thermo Fisher Scientific	Catalog # 13778150

**Deposited Data**		

Human XRCC4-DNA Ligase IV complex	Sibanda et al., 2001	PDB code: 1IK9
Human XLF	Li et al., 2008	PDB code: 2QM4
Human XRCC4-XLF complex	Wu et al., 2011	PDB code: 3W03
The N-terminal head domain of zebrafish SAS6	van Breugel et al., 2011	PDB code: 2Y3V
N-terminal head domain and beginning of coiled coil domain of Zebrafish SAS6	van Breugel et al., 2011	PDB code: 2Y3W
N-terminal domain of *C*. *elegans* SAS6	Hilbert et al., 2013	PDB code: 3PYI
N-terminal fragment of *L*. *major* SAS6	van Breugel et al., 2014	PDB code: 4CKP
Human PAXX	Ochi et al., 2015	PDB code: 3WTD
hCCCDC61^1-143^ structure	This paper	PDB code: 6HXT
zCCCDC61^1-168; F129E/D130A^ structure	This paper	PDB code: 6HXV
zCCCDC61^1-170^ structure	This paper	PDB code: 6HXY

**Experimental Models: Cell Lines**		

HEK293T	ATCC	ATCC: CRL-3216
RPE-1	Gift from Prof. Colin A. Johnson	N/A
RPE-1 PuroKO	Balmus et al., 2019	N/A
RPE-1 CCDC61 KO clone 1 and 2	this paper	N/A

**Experimental Models: Organisms/Strains**		

vfl3-1	Chlamydomonas Resource Center	CC-1686
vfl3-2	this paper	N/A

**Oligonucleotides**		

siRNA 1	Thermo Fisher Scientific	siRNA ID: s59736
siRNA 2	Thermo Fisher Scientific	siRNA ID: s59737
siRNA 3	Thermo Fisher Scientific	siRNA ID: s59738
Control siRNA	Thermo Fisher Scientific	siRNA ID: 4390084
*hCCDC61* knockout target sequence 1: GGAAGACGTAGTCCACCTGCAGG	This paper	N/A
*hCCDC61* knockout target sequence 2: GGAGCATGCCGTGCGGGTGATGG	This paper	N/A
RT-PCR primer forward: TGCAGCGATTTGGAGGATTT	This paper	N/A
RT-PCR primer reverse: CGGAGTTGGCCAGAGATTTC	This paper	N/A
Primers used for site-directed mutagenesis of human and zebrafish CCDC61, and human genomic DNA PCR in Table S2	N/A	N/A
Primers used to amplify *Chlamydomonas* VFL3 are listed in Table S3	N/A	N/A

**Recombinant DNA**		

hCCDC61	Synthesized by GenScript	UniProt: Q9Y6R9
zCCDC61	Source BioScience	IMAGE ID: 7406569. UniProt: Q08CF3
xCCDC61	Synthesized by Thermo Fisher Scientific	NCBI accession number: XP_018084688.1
PAXX	Ochi et al., 2015	N/A
GFP nanobody	Synthesized by GenScript	N/A
pGAT3-hCCDC61^1-143^	this paper	N/A
pGAT3-hCCDC61^1-143; F128E/D129A^	this paper	N/A
pSKB2LNB-zCCDC61^1-168; F129E/D130A^	this paper	N/A
Lipo-zCCDC61^1-170^	this paper	N/A
Lipo-zCCDC61^1-170; F129E/D130A^	this paper	N/A
pSKB2LNB-zCCDC61^146-280^	this paper	N/A
pSKB2LNB-zCCDC61^146-280; 5E^	this paper	N/A
pSKB2LNB-PAXX^1-137^-hCCDC61^144-287^	this paper	N/A
pSKB2LNB-PAXX^1-137^-hCCDC61^144-287; 5E^	this paper	N/A
pSKB2LNB-hSAS6^1-143^	this paper	N/A
pHAT5-GFP-nonobody	this paper	N/A
short-VFL3-TOPO	this paper	N/A
WT-VFL3-TOPO	this paper	N/A
pEGFP-C1-hCCDC61	this paper	N/A
pEGFP-C1-hCCDC61^F128E/D129A^	this paper	N/A
pEGFP-C1-hCCDC61^144-287-NES^	this paper	N/A
pEGFP-C1-hCCDC61^288-512^	this paper	N/A
pEGFP-C1-hCCDC61^1-457; F128E/D128A^	this paper	N/A
pEGFP-C1-hCCDC61^1-457; F128E/D129A/5E^	this paper	N/A
pcDNA3-3xHA-hCCDC61^1-457; F128E/D128A^	this paper	N/A
pcDNA3-3xHA-hCCDC61^1-457; F128E/D129A/5E^	this paper	N/A
pENTR-D-TOPO-xCCDC61	this paper	N/A
pCS2+-xCCDC61-RFP	this paper	N/A
pCS2+-Centrin2-BFP	this paper	N/A
pCS2+-Clamp-GFP	Park et al., 2008	N/A
AIO-GFP-hCCDC61	this paper	N/A
pGAT3	Peränen et al., 1996	Addgene: 112589
pHAT4	Peränen et al., 1996	Addgene: 112585
pHAT5	Peränen et al., 1996	Addgene: 112586
pSKB2LNB	Fekairi et al., 2009	N/A
pcEGFP-C1	Clontech	Catalog # 6084-1
pcDNA3	Invitrogen	Catalog # A-150228
AIO-GFP	Chiang et al., 2016	Addgene: 74119
pENTR-D-TOPO	Thermo Fisher Scientific	Catalog # K240020
pCR2.1-TOPO	Thermo Fisher Scientific	Catalog # K455001

**Software and Algorithms**		

Jpred	Drozdetskiy et al., 2015	http://www.compbio.dundee.ac.uk/jpred/
BackPhyre	Kelly and Sternberg, 2009	http://www.sbg.bio.ic.ac.uk/phyre2/html/page.cgi?id=index
HHPred	Söding et al., 2005	https://toolkit.tuebingen.mpg.de/tools/hhpred
PSI-BLAST	Altschul et al., 1997	https://blast.ncbi.nlm.nih.gov/Blast.cgi?CMD=Web&PAGE=Proteins&PROGRAM=blastp&RUN_PSIBLAST=on
MUSCLE	Edgar, 2004	https://www.drive5.com/muscle/
BOXSHADE	N/A	https://embnet.vital-it.ch/software/BOX_form.html
SIAS server	N/A	http://imed.med.ucm.es/Tools/sias.html
SeaView	Gouy et al., 2010	http://doua.prabi.fr/software/seaview
PhyML	Guindon et al., 2010	http://www.atgc-montpellier.fr/phyml/
FigTree	N/A	http://tree.bio.ed.ac.uk/software/figtree/
Modeller	Sali and Blundell, 1993	https://salilab.org/modeller/
TopMatch	Sippl and Wiederstein, 2012	https://topmatch.services.came.sbg.ac.at/
XDS	Kabsch, 2010	http://xds.mpimf-heidelberg.mpg.de/
CCP4 program suite	Winn et al., 2011	https://www.ccp4.ac.uk/ccp4i_main.php
iMOSFLM	Battye et al., 2011	Run from CCP4 program suite
Aimless	Evans, 2011	Run from CCP4 program suite
PHENIX suite	Adams et al., 2010	https://www.phenix-online.org/
MolProbity		Run from PHENIX suite
Coot	Emsley et al., 2010	https://www2.mrc-lmb.cam.ac.uk/personal/pemsley/coot/
PyMOL	N/A	https://pymol.org/2/
Consurf	Glaser et al., 2003	https://consurf.tau.ac.il/
SEDFIT	Schuck, 2003	http://www.analyticalultracentrifugation.com/sedfit.htm
Sedntrep	Dr Tomas Laue, University of New Hampshire	N/A
SEDPHAT	Schuck, 2003	http://www.analyticalultracentrifugation.com/sedphat/default.htm
GUSSI	Brautigam, 2015	http://biophysics.swmed.edu/MBR/software.html
Topspin	Bruker	N/A
SPARKY	T. D. Goddard and D. G. Kneller, University of California	https://www.cgl.ucsf.edu/home/sparky/
CRISPR DESIGN	Hsu et al., 2013	No longer available
LAS X	Leica	N/A
Zen	Zeiss	N/A
Volocity	Perkin Elmer	N/A
Fiji	Schindelin et al., 2012	https://imagej.net/Fiji/Downloads
Photoshop	Adobe	N/A
Huygens Professional	Scientific Volume Imaging	N/A
FCS EXPRESS 6	De Novo Software	N/A
Prism	GraphPad	N/A
Social Science Statistics	N/A	https://www.socscistatistics.com/tests/chisquare/

**Other**		

GSTrap FF 16/10	GE Healthcare Life Sciences	Catalog # 28936550
GSTrap HP	GE Healthcare Life Sciences	Catalog # 17528202
HisTrap HP	GE Healthcare Life Sciences	Catalog # 17524801
HisTrap FF	GE Healthcare Life Sciences	Catalog # 17525501
HiTrap Q HP	GE Healthcare Life Sciences	Catalog # 17115401
HiTrap Q FF	GE Healthcare Life Sciences	Catalog # 17515601
HiTrap Heparin HP	GE Healthcare Life Sciences	Catalog # 17040701
PD-10 desalting column	GE Healthcare Life Sciences	Catalog # 17085101
Superdex 75 16/600	GE Healthcare Life Sciences	Catalog # 28989333
Superdex S200 10/300	GE Healthcare Life Sciences	Catalog # 17517501
16 Chambered cover glass	Grace Bio-Labs	Catalog # 112358
Multi-spot slide	Thermo Fisher Scientific	Catalog # 9991090
400 mesh carbon-coated copper grids	Electron Microscopy Sciences	Catalog # CF400-Cu-50

### Resource Availability

#### Lead Contact

Further information and requests for resources and reagents should be directed to and will be fulfilled by the Lead Contact, Takashi Ochi (T.Ochi@leeds.ac.uk).

#### Materials Availability

All unique/stable reagents generated in this study are available from the Lead Contact without restriction.

#### Data and Code Availability

Coordinates and structure factors of crystal structures that are presented in this paper are available in the Protein Data Bank (PDB codes: 6HXT (hCCDC61^1-143^), 6HXV (zCCDC61^1-168;^ ^F129E/D130A^) and 6HXY (zCCDC61^1-170^)).

### Experimental Model and Subject Details

#### Human Cell Culture

All cells were grown in 37°C with 5% CO_2_. HEK293T cells (sex: female) were grown in D-MEM, GlutaMAX (Thermo Fisher Scientific) supplied with 10% FBS. RPE-1 cells (sex: female) were grown in D-MEM/F-12 supplied, GlutaMAX, sodium carbonate (Thermo Fisher Scientific) supplied with 0, 0.5 or 10% FBS, and 100 unit of penicillin and 100 μg/ml of streptomycin. RPE-1 and RPE-1 PuroKO that used in this study have been authenticated by STR profiling. STR profiling of HEK293T revealed a 68% match between our cells and the ATCC standard; this suggest a drift in our stock (which is fairly common for HEK293T), and thus these cells were used only for protein production (Figure S4E) and not for functional assays. In addition to these cell lines, RPE-1 PuroKO/CCDC61KO cells have been confirmed as mycoplasma free.

#### Xenopus Embryo Culture

*Xenopus* embryo were prepared as described previously (Hörmanseder et al., 2017). Briefly, mature *Xenopus laevis* males and females were obtained from Nasco. Females were injected with 50 units pregnant mare serum gonadotropin 3 days in advance and 500 units human chorionic gonadotropin 1 day in advance in the dorsal lymph sack to induce natural ovulation. Eggs were laid in a 1x MMR buffer (5mM HEPES pH 7.8, 100mM NaCl, 2mM KCl, 1mM MgSO4, 2mM CaCl2, 0.1mM EDTA). *Xenopus* embryos were cultured at 14°C in the 0.1x MMR until they reached stage 27/28. Our work with *Xenopus laevis* is covered under the Home Office Project License PPL 70/8591 and frog husbandry and all experiments were performed according to the relevant regulatory standard.

#### Chlamydomonas Culture

*Chlamydomonas reinhardtii* strains were maintained on solid Sager and Granick (R) growth medium at 25°C. For electroporation, *Chlamydomonas* cells were grown in Tris-acetate phosphate (TAP) medium at 25°C under constant illumination till the cell density reached 1∼3 x 10^6^ cells/ml. Transformants were selected on modified TAP medium (0.75 ml of Glacial acetic acid/1L TAP) supplied with 10 μg/ml hygromycin at 25°C. For immunofluorescence, *Chlamydomonas* cells were first resuspended in liquid M-N/5 medium for 4 hours and treated with autolysin for 30 min at 25°C before fixation of cells.

#### Bacterial Cell Culture

BL21(DE2) (New England Biolabs), C41(DE3) (Miroux and Walker, 1996) or Rosetta cells (a kind gift of Dr. John Kilmartin, MRC LMB, Cambridge, UK) were grown in LB or 2xTY media and used for protein expression and purification.

### Method Details

#### Amino-acid Sequence Analysis

As summarized in Figure S1A, hCCDC61 was identified as a paralog of XRCC4 using residue 1-213 of chain A of the crystal structure of XRCC4 (PDB code: 1IK9 (Sibanda et al., 2001)) as an input for the BackPhyre webserver (Kelley and Sternberg, 2009). Candidate proteins from BackPhyre whose alignments with XRCC4 covered its head domain were further analysed using HHpred (Söding et al., 2005) and JPred (Cole et al., 2008). Criteria to be defined as XRCC4-like proteins were: a) the candidates were predicted as XRCC4-superfamily members using HHPred and b) they have secondary-structure profiles that are similar to these family members.

Orthologs of hCCDC61 (UniProt accession number: Q9Y6R9) were identified using PSI-BLAST (Altschul et al., 1997) and aligned using MUSCLE (Edgar, 2004) on SeaView (Gouy et al., 2010). A phylogenetic tree was created using the PhyML server (Guindon et al., 2010) with the default setting and 100 bootstrap replicates, and edited using FigTree (http://tree.bio.ed.ac.uk/software/figtree/). Secondary structure predictions were carried out using the JPred webserver.

The crystal structure of zebrafish SAS6 (PDB code: 2Y3W (van Breugel et al., 2011)) was used as a template to model a structure of human SAS6 using Modeller (Sali and Blundell, 1993). This model together with crystal structures of hCCDC61 (from this study), XRCC4, XLF and PAXX (PDB codes: 1IK9 (Sibanda et al., 2001), 2QM4 (Li et al., 2008) and 3WTD (Ochi et al., 2015) respectively) were superposed and aligned using TopMatch (Sippl and Wiederstein, 2012). Alignments were manually adjusted using SeaView.

#### Constructs and Antibodies

The human *CCDC61* gene was codon optimized for *E*. *coli* and synthesized (GenScript), that of *Danio rerio* was purchased from Source BioScience and that of *Xenopus laevis* was synthesized without codon optimization (Thermo Fisher Scientific). *hCCDC61* constructs were PCR amplified and cloned into a pGAT3 (Peränen et al., 1996) or pSKB2LNB vector (a pET28-derived vector resulting in a fusion protein containing a N-terminally, PreScission protease-cleavable His_6_ tag) (Fekairi et al., 2009) for bacterial expression and pEGFP-C1 or pcDNA3-3xHA for human expression. NES peptides (LQLPPLERLTLD (Wen et al., 1995)) were added to some of *gfp-hCCDC61* constructs after short linkers (SGSS) by PCR. The z*CCDC61* constructs were cloned into pSKB2LNB or a bacterial-expression plasmid encoding a N-terminal His-tagged lipoyl domain from *Bacillus stearothermophilus* dihydrolipoamide acetyltransferase for bacterial expression. The *xCcdc61* gene was cloned into pENTR-D-TOPO vector to fuse it to RFP in pCS2+ vectors. Plasmids expressing Centrin2 and Clamp were kind gifts from Dr John Wallingford (Park et al., 2008). The *Centrin2* gene followed by a *bfp* gene was cloned into the pCS2+ vector. The plasmid containing the *bfp* gene was a kind gift from Dr Keith Boyle (MRC LMB). A GFP-nanobody gene was codon optimized for *E*.*coli* and synthesized (GenScript) and cloned into pHAT5 vector (Peränen et al., 1996). Site-directed mutagenesis was carried out by PCR using forward and reverse primers encoding mutant amino acids (Table S2).

The ∼4 kb *Chlamydomonas VFL3* gene, which includes ∼600 bp upstream of the start codon and ∼700 bp downstream of the stop codon, was amplified by PCR (VFL3-1F and VFL3-7R) using the CloneAmp HiFi Premix (Clontech) and cloned into the pCR2.1-TOPO vector (Thermo Fisher Scientific). No rescue of *vfl3-1* was observed with this 8 Kb VFL3-TOPO plasmid (short-VFL3-TOPO), presumably due to short promoter at the 5’ end. Therefore, a ∼3.6 kb fragment upstream of *VFL3*, which include the 5’ UTR and part of exon 1 of *VFL3*, was amplified by PCR (CloneAmp HiFi, VFL3-NotI-F and VFL3-NdeI-R) and cloned into the short-VFL3-TOPO plasmid digested with *Not*I and *Nde*I by Infusion HD cloning (Clonetech). This plasmid (WT-VFL3-TOPO) contains ∼3.6 kb upstream of the *VFL3* start codon and it rescues the *vfl3-1* mutant phenotype.

To generate the 3x HA tagged *VFL3* plasmids, a *Hpa*I restriction site was introduced in-frame to either exon 7 or exon 9 via overlapping PCR by creating the restriction enzyme site in the primers. For the exon 7-HpaI plasmid, a 1.5 kb fragment was amplified by primers VFL3-13F-AflII and VFL3-13R-HpaI and a 0.2 kb fragment was amplified by primers VFL3-14F-HpaI and VFL3-14R-SalI from the short-VFL3-TOPO plasmid. These two fragments were gel purified and used as templates in a second round of PCR using primers VFL3-13F-AflII and VFL3-14R-SalI for a 1.7 kb fragment. This fragment was digested with *Afl*II and *Sal*I and replaced the corresponding fragment from the short-VFL3-TOPO plasmid. The HA epitope tag was amplified by PCR (exon 7-HpaI-HA-F and R) and cloned into the *Hpa*I digested plasmid via Infusion HD cloning. For the exon 9-HpaI plasmid, a 0.8 kb fragment was amplified by primers VFL3-7F and VFL3-15R-HpaI and a 0.4 kb fragment was amplified by primers VFL3-15F-HpaI and VFL3-3R from the short-VFL3-TOPO plasmid. A second round PCR was used to amplify a 1.2 kb fragment with VFL3-7F and VFL3-3R. This fragment was digested with *Sal*I and *Pml*I and replaced the corresponding fragment from the short-VFL3-TOPO plasmid. The HA epitope tag was amplified by PCR (exon 9-HpaI-HA-F and R) and cloned into the *Hpa*I digested plasmid via Infusion HD cloning. The ∼3.6 kb upstream fragment described above was then introduced the exon 7-HA and exon 9-HA plasmids respectively to create exon 7-HA-VFL3 and exon 9-HA-VFL3 plasmids used in *vfl3-2* rescue. A similar strategy was used to introduce a GFP-tag (Fuhrmann et al., 1999) in-frame to exon 7 of VFL3 at the *Hpa*I site. The *UNI2*::*HA* gene was introduced into the *vfl3* strains by crosses so that only one integration site was present in all of the strains.

Both FD-VFL3 and 5E-VFL3 plasmids were generated by overlapping PCR. For the FD-VFL3 plasmid, a 0.4 kb fragment was amplified by primers VFL3-8F and VFL3-FD-R and a 0.7 kb fragment was amplified by primers VFL3-FD-F and VFL3-5R from the short-VFL3-TOPO plasmid. These two fragments were gel purified and used as templates in a second round of PCR using primers VFL3-8F and VFL3-5R for a 1.1 kb fragment. This fragment was digested with *Afl*II and *Bbv*CI and replaced the corresponding fragment from the exon 7-HA-VFL3 plasmid. For the 5E-VFL3 plasmid, a 0.6 kb fragment was amplified by VFL3-2F and VFL3-5E-R and a 1.1 kb fragment was amplified by primers VFL3-5E-F and VFL3-6R from the short-VFL3-TOPO plasmid. These two fragments were gel purified and used as templates in a second round of PCR using primers VFL3-2F and VFL3-6R for a 1.7 kb fragment. This fragment was digested with *Bbv*CI and *Sal*I and replaced the corresponding fragment from the exon 7-HA-VFL3 plasmid. All primers used to amplify *Chlamydomonas VFL3* are listed in Table S3.

The following primary antibodies were used: ARL13B (Proteintech, 17711-1-AP) 1/500 for immunofluorescent staining (IF), α-tubulin (Sigma-Aldrich, T9026) 1/500 for IF, acetylated α-tubulin antibody (Abcam, ab179484) 1/500 for IF, γ-tubulin (Sigma-Aldrich, T6557) 1/1000 for IF, GFP (Abcam, ab13970 or Thermo Fisher Scientific, 3E6, A11120) 1/2000 or 1/250 for IF, HA (a kind gift from Dr. Manu Hedge or Roche, 3F10, 11867423001) 1/200 for IF or 1/3000 for WB, centrin (a kind gift from Dr. Jeffrey L. Salisbury) 1/250 for IF, and Centrin 3 (Abnova, H00001070-M01) 1/500 for IF. Secondary antibodies used include Alexa-488-conjugated Donkey anti-rabbit (Thermo Fisher Scientific, A21206) 1/2000 for IF, Alexa-488-conjugated Goat anti-chicken (Thermo Fisher Scientific, A11039) 1/2000 for IF, Alexa-488-conjugated goat anti-rat antibody (Invitrogen, API83P) 1/500 for IF, Alexa-488-conjugated goat anti-mouse antibody (Molecular probes, A11001) 1/500 for IF, Alexa-555-conjugated Goat anti-mouse (Thermo Fisher Scientific, A21422) 1/2000 for IF, Alexa 594-conjugated chicken anti-mouse antibody (Invitrogen, A21201) 1/500 for IF, Alexa 594-conjugated goat anti-rabbit antibody (Molecular probes, A11037) 1/500 for IF, and Alexa 647-conjugated goat anti-mouse antibody (Thermo Fisher Scientific, A21235) 1/500 for IF, ATTO 647N-conjugated goat anti-rabbit antibody (Sigma-Aldrich , 40839) 1/2000 for IF and HRP-conjugated mouse anti-rabbit antibody (Santacruz Biotechnology, sc-2357) 1/3000 for WB.

#### Analysis of Chlamydomonas Transcripts

For *Chlamydomonas* RNA isolation, ∼5x10^8^ cells were resuspended in 10 ml nitrogen-free medium (M-N/5) for 4 hours at 25°C. The cells were collected at 500 g for 5 minutes at room temperature and the pellet was resuspended in 1 ml of Buffer RLT (reagent from Qiagen RNeasy Mini Kit) with 10 μl of 2-mercaptoethanol. Cells were homogenized by passing through a 20-gauge needle 20 times and centrifuged at 11000 g for 5 minutes at room temperature. The supernatant was collected and RNA extraction was performed with the RNeasy Mini Kit (Qiagen) according to manufacturer’s recommendation. Five micrograms of total RNA from each strain was treated with 5 U of RNase-free DNase I (Thermo Fisher Scientific) in 50 μl reaction at 37°C for 30 minutes. The reaction was terminated by addition of 5 μL 50 mM EDTA and heat inactivation at 65°C for 10 min. One microgram of DNase-treated RNA from each strain were added to SuperScript IV VILO Master Mix (Thermo Fisher Scientific). The reverse transcription reactions were performed according to manufacturer’s recommendation with the reverse transcription temperature set to 60°C.

#### Protein Purification

Purification of hCCDC61^1-143^ was carried out as follows. BL21(DE3) cells (New England Biolabs) that carried the pGAT3-*hCCDC61*^*1-143*^ plasmid were grown in LB media at 37°C till OD_600_ reached between 0.6-1.0, and the gene expression was induced by 0.5 mM IPTG after cooling the cell cultures to 16°C. The cell cultures were grown at the temperature overnight. Collected cells were suspended in 8 ml of a lysis buffer (50 mM Tris-HCl pH 8.0, 300 mM NaCl, 1 mM EDTA, 5 mM DTT, 1x cOmplete Protease Inhibitor Cocktail EDTA-free (Roche)) per gram of cells. The lysis was carried out by sonication. Cell debris were removed by centrifuging at 32,000 g for 45 min at 4°C. The supernatant after the centrifugation was collected and loaded onto a GSTrap FF 16/10 column (GE Healthcare) equilibrated with the lysis buffer without the protease inhibitor. After washing the column with the lysis buffer, bound molecules were eluted with the same equilibrated buffer but 25 mM reduced L-glutathione. The elution was dialyzed against 5L of 30 mM Tris-HCl pH 8.0 150 mM NaCl, 0.5 mM EDTA, 5 mM 2-mercaptoethanol at 4°C overnight after tev protease was added. The cleaving of the His-GST tag leaves the extra amino-acid sequence Gly-Ser at the N-terminus of hCCDC61^1-143^. The dialyzed sample was further dialyzed against 5L of 30 mM Tris-HCl pH 8.0 at 4°C, 150 mM NaCl, 20 mM imidazole, 2 mM 2-mercaptoethanol at 4°C for four hours. The sample was loaded onto a 5ml HisTrap HP column (GE Healthcare), and the flow through and the first 5ml wash were collected. The collected sample was diluted three-fold with 20 mM Tris-HCl pH 8.0, 2 mM DTT and loaded onto a 5ml HiTrap Q HP column (GE Healthcare) equilibrated with 20 mM Tris-HCl pH 8.0, 50 mM NaCl, 2 mM DTT. The bound molecules were eluted with a linear gradient to 600 mM NaCl. Peak fractions containing hCCDC61^1-143^ were collected and concentrated to 2.5 ml to load onto a PD-10 column (GE Heathcare) equilibrated with 20 mM Tris-HCl pH 8.0, 150 mM NaCl, 2%(v/v) glycerol, 2 mM DTT. Purified hCCDC61^1-143^ was concentrated and stored at -80°C after snap-freezing in liquid nitrogen (Figure S7D). hCCDC61^1-143; F128E/D129A^ mutant and SeMet replaced hCCDC61^1-143^ were purified in a similar way.

Purification of zCCDC61^1-168; F129E/D130A^ was carried out as follows. The supernatant of crude bacterial extracts containing zCCDC61^1-168; F129E/D130A^ was prepared in a similar way to that of hCCDC61^1-143^. However, we used C41 cells (Miroux and Walker, 1996) instead of BL21(DE3) and 50 mM Tris-HCl pH 8.0, 300 mM NaCl, 10 mM imidazole, 2 mM 2-mercaptoethanol, 1 mM AEBSF. 5ml of Ni-NTA resin (Expedion) were added to the extracts and incubated at 4°C for 120 min. The resin was washed with 50 mM Tris-HCl pH 8.0, 300 mM NaCl, 10 mM imidazole, 2 mM 2-mercaptoethanol and the same buffer but 30 mM imidazole. Bound molecules were eluted with the same buffer but 300 mM imidazole. The GST-PreScission protease and EDTA at the final concentration of 0.5 mM were added to the elution, which was dialyzed against 5L of 30 mM Tris-HCl pH 8.0, 150 mM NaCl, 0.5 mM EDTA, 2 mM 2-mercaptoethanol at 4°C overnight. The cleaving the His tag leaves the extra amino-acid sequence Gly-Pro-His at the N-terminus of zCCDC61^1-168; F129E/D130A^. 0.5 ml of glutathione sepharose 4B were added to the dialyzed sample and incubated at 4°C for 60 min. The supernatant was collected, diluted three-fold with 20 mM Tris-HCl pH 8.0, 2 mM DTT and loaded onto a 5 ml HiTrap Q HP column equilibrated with 20 mM Tris-HCl pH 8.0, 50 mM NaCl, 2 mM DTT. Bound molecules were eluted with a linear gradient of 400 mM NaCl. Peak fractions containing zCCDC61^1-168; F129E/D130A^ were collected. The buffer of the sample was exchanged to 20 mM Tris-HCl pH8.0, 100 mM NaCl, 2 mM DTT by a PD-10 column and the protein stored at -80°C after concentration (Figure S7D).

Construct zCCDC61^1-170^ fused to the C-terminus of a lipoyl-domain tag (Lipo-zCCDC61^1-170^) (for SEC-MALS analysis) and its F129E/D130A mutant (for SEC-MALS analysis) were expressed in *E*. *coli* C41 in 2xTY and purified by Ni-NTA (Qiagen) beads using standard methods. Subsequently, eluates were subjected to a size exclusion chromatography step in 10 mM Tris-HCl, pH 8.0, 50 mM NaCl, 2 mM DTT and the purifications finished by ion-exchange chromatography on a HiTrap Q-FF (GE Healthcare) column using a linear salt gradient from 10 mM Tris-HCl, pH 8.0, 2 mM DTT to 10 mM Tris-HCl, pH 8.0, 2 mM DTT, 1 M NaCl. Proteins were concentrated and snap frozen in liquid nitrogen and stored at -80°C. To purify zCCDC61^1-170^ without the lipoyl-domain tag, Lipo-zCCDC61^1-170^ was incubated with the tev protease after the Ni-affinity purification step. Cleaving the tag leaves the extra amino-acid sequence Gly-Gly-Ser at the N-terminus of zCCDC61^1-170^. The zCCDC61^1-170^ solution was loaded onto a HisTrap FF column to remove the tag and tev protease and then loaded onto a HiTrap Q HP column after being diluted to 175 mM NaCl concentration by 20 mM Tris-HCl pH 8.0. The flow through fractions containing zCCDC61^1-170^ were collected and loaded onto a PD-10 buffer exchange column equilibrated with 10 mM Tris-HCl pH8.0, 300 mM NaCl, 2 mM DTT and concentrated before being snap-frozen in liquid nitrogen. Lipo-zCCDC61^1-170; F129E/D130A^ used for Figure 2D was purified in a similar way.

Purification of zCCDC61^146-280^ was carried out as follows. A Ni-NTA affinity purification of zCCDC61^146-280^ was carried out in a similar way to that of zCCDC61^1-168; F129E/D130A^ but using 500 mM NaCl in the purification buffers. GST-PreScission protease and EDTA (at a final concentration of 0.5 mM) were added to the elution. Cleaving the tag leaves the extra amino acid sequence Gly-Pro-His-Asn at the N-terminus of the protein. 1 ml of glutathione sepharose 4B were added to the sample and incubated at 4°C for 60 min. The supernatant was collected and diluted 3-to-5 with 30 mM Tris-HCl pH 8.0, 5 mM DTT. The diluted sample was loaded on to a 5 ml HiTrap Q HP equilibrated with 30 mM Tris-HCl pH8,0, 300 mM NaCl, 5 mM DTT. The flow through was collected and concentrated to ∼3 ml before loading onto a Superdex 75 16/600 column (GE Healthcare) equilibrated with 20 m HEPES pH 7.5, 500 mM NaCl, 2 mM DTT. Fractions containing zCCDC61^146-280^ were collected and diluted two-fold with 20 mM HEPES pH 7.5 before loading onto a 5 ml HiTrap Heparin HP column equilibrated with 20 m HEPES pH 7.5, 200 mM NaCl, 2 mM DTT. Bound molecules were eluted with a linear gradient of 20 m HEPES pH7.5, 1 M NaCl, 2 mM DTT. Fractions containing zCCDC61^146-280^ were collected, and the buffer of the protein was exchanged to 20 mM HEPES pH 7.5, 200 mM NaCl, 2 mM DTT using a PD-10 column. The sample was concentrated to a desired concentration and snap frozen in liquid nitrogen before storing at -80°C (Figure S7D). zCCDC61^146-280; 5E^ mutant was purified in a similar way to zCCDC61^146-280^, but a HiTrap Q HP column was used instead of the HiTrap Heparin HP column. The protein has the extra amino-acid sequence Gly-Pro-His-Asp at its N-terminus.

Purification of hCCDC61^288-512^ was carried out in a similar manner to zCCDC61^1-170^. hCCDC61^288-512^ fused to the C-terminus of a lipoyl-domain tag was expressed in *E*. *coli* C41. All following steps were carried out at room temperature because hCCDC61^288-512^ tends to precipitate at 4°C. After the Ni-NTA step, the eluted proteins were loaded onto a 5ml HiTrap Heparin HP column (GE Healthcare) equilibrated with 20 mM HEPES pH 7.5, 300 mM NaCl, 2 mM DTT. Bound proteins were eluted with a linear gradient of 20 mM HEPES pH 7.5, 1 M NaCl, 2 mM DTT. Fractions containing hCCDC61^288-512^ were collected and the lipoyl tag was cleaved by adding tev protease, which leaves the extra amino-acid sequence Gly-Gly-Ser at the N-terminus of hCCDC61^288-512^. The cleaved sample was passed onto a 5 ml HisTrap HP column. The flow through was collected and dialyzed against 3L of 20 mM HEPES pH 7.5, 500 mM NaCl, 2 mM DTT. The dialysed sample was concentrated and stored at -80°C after snap frozen in liquid nitrogen (Figure 3C).

To stabilise hCCDC61^144-287^ and hCCDC61^144-287; 5E^, both constructs were fused to the C-terminus of residue 1-137 of PAXX (Ochi et al., 2015) and cloned into pSKB2LNB vector. The proteins were expressed and purified using Ni-NTA as described above. Eluted proteins were cleaved with the GST-PreScission protease and dialyzed in 2L of 30 mM Tris-HCl pH 8.0, 2 mM 2-mercaptoethanol overnight supplied with 500 mM NaCl (hCCDC61^144-287^) and 200 mM NaCl (hCCDC61^144-287; 5E^) at 4°C. The cleavage leaves the extra amino-acid sequence Gly-Pro-His at the N-terminus of these constructs. As for hCCDC61^144-287^, the dialyzed sample was diluted two-fold with 20 mM Tris-HCl pH8.0, 2 mM DTT and loaded on to tandemly connected 5 ml GSTrap and HiTrap Q HP equilibrated with 30 mM Tris-HCl pH 8.0, 300 mM NaCl, 2 mM DTT. The flow through was loaded onto a 5 ml HiTrap Heparin HP column equilibrated with 20 mM HEPES pH 7.5, 200 mM NaCl, 2 mM DTT. Bound proteins were eluted with a linear gradient of 20 mM HEPES pH7.5, 1 M NaCl, 2 mM DTT. The fractions containing hCCDC61^144-287^ were collected and passed onto a PD-10 column equilibrated with 20 mM HEPES pH 7.5, 500 mM NaCl, 2 mM DTT. The purified protein was concentrated and stored at -80°C after snap freezing in liquid nitrogen (Figure 3C). As for hCCDC61^144-287; 5E^, the dialyzed sample was diluted two-fold with 50 mM HEPES pH 7.5, 5 mM 2-mercaptoethanol and loaded onto a 5 ml GSTrap column. The flow through was loaded onto a 5 ml HiTrap Q HP column equilibrated with 20 mM HEPES pH 7.5, 100 mM NaCl, 2 mM DTT. Bound proteins were eluted with a linear gradient of 20 mM HEPES pH 7.5, 1 M NaCl, 2 mM DTT. The fractions containing hCCDC61^144-287; 5E^ were collected and diluted with 20 mM HEPES pH 7.5, 200 mM NaCl, 2 mM 2-mercaptoethanol. The diluted sample supplied with 20 mM imidazole was loaded onto a 5 ml HisTrap HP column, and the flow through was collected. The protein was concentrated and diluted with 20 mM HEPES pH7.5, 200 mM NaCl, 2 mM DTT. The procedure was repeated three times. Finally, the concentrated sample was stored at -80°C after snap freezing in liquid nitrogen (Figure S4D).

For purification of ^15^N-labelled human SAS6, DNA encoding human *SAS6*^*1-143*^ was cloned into pSKB2LNB vector. This construct was expressed in *E*. *coli* Rosetta in minimal medium containing ^15^NH_4_Cl and purified by standard methods using Ni-NTA (Qiagen) chromatography. The eluate was dialyzed (in the presence of GST-PreScission protease) against 10 mM Tris-HCl, pH 8.0, 2 mM DTT and further purified by ion-exchange chromatography on a HiTrap Q-FF (GE Healthcare) column using a linear salt gradient from 0 mM to 1 M NaCl in 10 mM Tris-HCl, pH 8.0, 2 mM DTT followed by size exclusion chromatography in 20 mM Tris-HCl, pH 8.0, 150 mM NaCl, 2 mM DTT (Figure S7D).

GFP nanobody was purified based on a published protocol (Kubala et al., 2010). BL21(DE3) cells that carried the pHAT5-GFP-nanobody plasmid were grown in 6L of LB media at 37°C till OD_600_ reached between 0.6-1.0, and the gene expression was induced by 1 mM IPTG after cooling the cell cultures to 16°C. The cell cultures were grown at the temperature overnight. Collected cells were suspended in 5 ml of a lysis buffer (50 mM Tris-HCl pH 8.0, 300 mM NaCl, 5%(v/v) glycerol, 0.1%(v/v) NP-40, 10 mM imidazole, 1x cOmplete Protease Inhibitor Cocktail EDTA-free) per gram of cells. The lysis was carried out by sonication. Cell debris were removed by centrifuging at 27,000 g for 30 min at 4°C. The supernatant after the centrifugation was collected and loaded onto 2x 5ml HisTrap HP columns (GE Healthcare) equilibrated with the lysis buffer without the protease inhibitor. The column was washed with 50 mM HEPES pH7.5, 1 M NaCl, 0.2%(v/v) NP-40, 50 mM imidazole and 50 mM HEPES pH7.5, 500 mM NaCl, 50 mM imidazole. The bound molecules were eluted with 50 mM HEPES pH7.5, 500 mM NaCl, 300 mM imidazole. The elution was dialyzed against 5L of 0.2 M NaHCO_3_ pH8.3, 500 mM NaCl at 4°C overnight. Dialyzed GFP nanobody was concentrated and stored at -80°C after snap-freezing in liquid nitrogen.

#### Protein Crystallization

SeMet hCCDC61^1-143^ was crystallized at 20°C in a hanging drop containing 10 mg/ml of the protein and 8% (w/v) PEG6,000, 100 mM Tris-HCl pH 7.3 in a 1:1 ratio. zCCDC61^1-168; F129E/D130A^ was crystallized at 20°C in a sitting drop containing 10 mg/ml of the protein and 8% (w/v) PEG 6,000, 100 mM Bicine pH 9.0, 3% Trimethylamine N-Oxide in a 1:1 ratio. zCCDC61^1-170^ was crystallized at 20°C in a sitting drop containing 4.62 mg/ml of the protein and 100 mM Citric acid pH 5.0, 1 M LiCl in a 1:1 ratio. Single crystals of the proteins were dipped into cryo-protection solutions, which were 70% reservoir and 30% ethylene glycol, and flash frozen in liquid nitrogen.

#### X-ray Crystallography

Diffraction images of the crystals were collected at I02 in Diamond Light Source (DLS) for SeMet hCCDC61^1-143^, at MRC LMB using an in-house X-ray diffraction machine (RIGAKU FR-E+ SuperBright) for zCCDC61^1-168; F129E/D130A^ and at I03 in DLS for zCCDC61^1-170^. The collected data were indexed and integrated using XDS (Kabsch, 2010) for the SeMet hCCDC61^1-143^ data, iMOSFLM (Battye et al., 2011) for zCCDC61^1-168;F129E/D130A^ and zCCDC61^1-170^ data, and scaled using Aimless (Evans, 2011), which were run from CCP4 program suite (Winn et al., 2011). The phenix.autosol module of PHENIX suite (Adams et al., 2010) was used to calculate phases for structure factors of the SeMet hCCDC61^1-143^ data by the SAD method. Phases for structure factors of the zCCDC61^1-168; F129E/D130A^ or zCCDC61^1-170^ data were determined by the phenix.phaser module using the structure of SeMet hCCDC61^1-143^ or zCCDC61^1-168; F129E/D130A^ as a probe for molecular replacement respectively. The initial structures were build using the phenix.autobuild module. The models were refined manually using Coot (Emsley et al., 2010) and computationally using the phenix.refine module until no further improvements of the map were observed. TLS groups were selected as each chain for hCCDC61^1-143^ and as each chain divided into two groups (from N-terminus to residue 144 and from 145 to the C-terminus) for zCCDC61^1-168; F129E/D130A^ and zCCDC61^1-170^. Non-crystallographic symmetry restraints were not applied for the refinement of these structures. For the refinement of zCCDC61^1-170^, E129 and A130 of zCCDC61^1-168; F129E/D130A^ were replaced with F129 and D130, and the model was refined as described above. The final structural models were validated using MolProbity (Williams et al., 2018) run from PHENIX suite. All protein-structure graphics were produced using PyMOL (Schrödinger, 2015).

#### Analytical Ultracentrifugation

hCCDC61^1-143^ and hCCDC61^1-143; F128E/D129A^ at approximately 480 μM (7.9 mg/ml) in 20 mM Tris-HCl pH 8.0, 150 mM NaCl, 2 mM DTT were subjected to velocity sedimentation at 50,000 rpm at 4°C in an An50Ti rotor using an Optima XL-I analytical ultracentrifuge (Beckmann). The data were analysed in SEDFIT 15.0 (Schuck, 2003) using a c(s) distribution model. The partial-specific volumes (v-bar), solvent density and viscosity were calculated using Sednterp (Dr Thomas Laue, University of New Hampshire). To determine the dissociation for dimerization, *K*_d,_ of hCCDC61^1-143^ homodimer, 110 μL with total protein concentrations of 12, 4 and 1.3 mg/ml were loaded in 12 mm 6-sector cells and centrifugated at 11,600, 19,700 and 34,000 rpm at 4°C until equilibrium had been reached. Data were processed and analyzsed using SEDPHAT 13b (Schuck, 2003). Data were plotted with the program GUSSI (Brautigam, 2015).

#### SEC-MALS

The mass and hydrodynamic radius of CCDC61 constructs in solution was determined using SEC-MALS as described previously (van Breugel et al., 2011). SEC was in 10mM Tris-HCl pH 7.4, 150mM NaCl (1, 6.5 and 65 mg/ml of His_6_-lipoyl-zCCDC61^1-170^ and 1, 6.8 and 73 mg/ml of its F129E/D130A mutant). SEC used a Superdex S200 10/300 column (GE Healthcare) running at 0.5 ml/min. The concentrations quoted are at loading and these will be at least 10 times lower during chromatography due to dilution on the column. Experiments were performed at room temperature. Since the coiled-coil regions present in His_6_-lipoyl-zCCDC61^1-170^ and its mutant are very short (∼20 amino acids), coiled-coil dimerization is inefficient, explaining the presence of monomer species in the SEC-MALS runs.

#### Circular Dichroism (CD)

Purified zCCDC61^146-280^ and its 5E mutant were dialyzed against 1L of 20 mM sodium phosphate pH7.5, 500 mM NaCl, 1 mM TCEP at 4°C overnight and adjusted to a concentration of 0.375 mg/ml. The CD measurement was done at 5°C. Far-UV CD spectra at 5°C and thermal melts at 222 nm were measured using a Jasco J815 spectropolarimeter (JASCO (UK) Ltd) in 20 mM sodium phosphate pH7.5, 500 mM NaCl, 1 mM TCEP. Following dialysis into this buffer, samples of zCCDC61^146-280^ and its 5E mutant were diluted to 0.375 mg/ml and measured in a 1 mm pathlength cuvette. Thermal melts were performed at a heating rate of 1C°/min.

#### NMR

NMR data were collected at 20°C on a Bruker Avance II+ 700 MHz spectrometer, equipped with a cryogenic triple-resonance TCI probe. 2D ^1^H,^15^N BEST-Trosy data sets for 56 μM of ^15^N-labelled hSAS6^1-143^ on its own and in the presence of 48 μM of hCCDC61^1-143^ were acquired in 20 mM Tris pH8.0, 150 mM NaCl and 2 mM DTT. Data were processed using Topspin 3.0 (Bruker) and analyzed using SPARKY (T. D. Goddard and D. G. Kneller – University of California, San Francisco).

#### Microtubule Pelleting Assay

Taxol-stabilized microtubules were prepared as described on Anthony Hyman’s lab website (http://hymanlab.mpi-cbg.de/hyman_lab/wp-content/uploads/2012/08/Tubulin-Protocols-Mitchison.pdf). All centrifugation steps were carried out in 7 x 20 mm polycarbonate-centrifuge tubes using a TLA100 rotor and Optima TL ultracentrifuge (Beckman Culture). 20 μM Tubulin purified from pig brains (a kind gift of Dr. Andrew Carter, MRC LMB, Cambridge, UK) were polymerized at 37°C in BRB80 (80 mM PIPES pH 6.8, 1 mM MgCl2, 1 mM EGTA) supplied with 1 mM GTP and 1 mM DTT by adding 1/10 volume of 2, 20 and 200 μM of Taxol stepwise. Taxol-stabilized microtubule were pelleted by centrifugation at 70,000 rpm for 12 min at 25°C. The microtubule pellets were resuspended in the reaction buffer (20 mM Tris-HCl pH7.4, 200 mM NaCl, 1 mM DTT, 20 μM Taxol). In order to find an optimal tubulin / subtilisin ratio for removing the tubulin C-termini, we first mixed 2 mg/ml tubulins with a four-fold dilution series of subtilisin A (Sigma-Aldrich) starting from 1:1 weight ratio. This experiment was performed at 37°C for 15 min. The digestion reaction was terminated by adding 10 mM PMSF and incubated at 37°C for 5 min before being centrifuged to pellet microtubules. 20 μM of CCDC61 constructs in the reaction buffer were spun at 70,000 rpm for 12 min at 25°C. 45 μl of the supernatant of each construct were mixed with the equal volume of 20 μM of the stabilized microtubules in reaction buffer. The mixed samples were incubated at RT for 15 min. 85 μl of the mixed samples were centrifuged through a cushion of 50 μl of the reaction buffer supplied with 40%(v/v) glycerol at 70,000 rpm for 30 min at 25°C. The supernatants and pellets were analyzed using SDS-PAGE. Mixed samples before the ultracentrifugation step were also used for negative-stain EM visualization.

#### Knockout and Knockdown of hCCDC61

Knockout of CCDC61 in RPE-1 PuroKO cells (Balmus et al., 2019) was done using methods and reagents as described before (Chiang et al., 2016). Briefly, two target sites in exon 1 of *CCDC61* (5'-GGAAGACGTAGTCCACCTGCAGG-3' and 5'-GGAGCATGCCGTGCGGGTGATGG-3') of Cas9 were designed by CRISPR DESIGN (Hsu et al., 2013). The all-in-one plasmid encoding these sites (AIO-GFP-hCCDC61) was transfected to RPE-1 cells by electroporation using the NEON transfection system (Thermo Fisher). After 48 hours, GFP-positive cells were FACS sorted in three 96-well plates at the Cambridge Stem Cell Institute, University of Cambridge. Cells were incubated at 37°C in 5% CO_2_ about a month till they became confluent. Genomic DNAs of the cells were extracted using QuickExtract DNA extraction solution (Cambio) and subjected to PCR using two primers (5'-TTCCAGGGTTCCATGGGTCTAGGTTTCTCTCTCATCTCCTT

-3' and 5'-CGAGGTCGACGAATTCGGCACACTCACAGCCAGCATCGAA

-3'). The PCR products were cloned into a pHAT4 (Peränen et al., 1996) vector to be sequenced. Two clones that had inserts causing premature stop codons in both alleles of the exon (Figure S5A) were selected for further studies. For counting of the centriole number, parental and CCDC61 knockout cell lines were treated with 100 μM monastrol (Sigma-Aldrich) for 4 hours. For ciliation assay, cells were serum starved in media containing 0.5% FBS for 24 and 48 hours. To determine proliferation kinetics, cells were seeded in 12 well plate at 5x10^3^ cells/well and real-time quantitative live cell analysis was carried out for 96 hours using IncuCyte ZOOM (Essen BioScience), imaging 9 positions per well every 3 hours.

Knockdown of CCDC61 was carried out by transfecting three different Silencer Select siRNAs (siRNA IDs: s59736 as siRNA 1, s59737 as siRNA 2 and s59738 as siRNA 3) (Life Technologies) or (Ambion control siRNA) to RPE-1 cells. Briefly, RPE-1 cells were transfected with Lipofectamine RNAiMAX reagent (Thermo Fischer Scientific). The siRNAs were used at a final concentration of 60 nM and the siRNA treatments were carried out for 72 hours after transfection. To assess ciliation, 48 hours after siRNA transfection RPE-1 cells were serum starved in media containing 0.5% FBS for 24 hours. Knockdown efficiencies were assessed by reverse transcription PCR (RT-PCR) using a forward primer (5'-TGCAGCGATTTGGAGGATTT-3') and a reverse primer (5'-CGGAGTTGGCCAGAGATTTC-3').

#### Fluorescent and Immunofluorescent Microscopy

GFP-hCCDC61 constructs were transfected to RPE-1 cells using Lipofectamine 3000 (Thermo Fisher Scientific) by mixing 500 ng of each pEGFP-C1-*hCCDC61* construct with 1 μl of P3000 reagent and 1 μl of Lipofectamine 3000 reagent in Opti-MEM (Thermo Fisher Scientific) before adding the mixture to the cells grown on a coverslip in a well of a 24-well plate. After 24 hours of transfection, cells were fixed with 4% (w/v) formaldehyde for fluorescent microscopy or cold methanol and stained with antibodies for immunofluorescent microscopy as indicated in the main text. ProLong Diamond Antifade Mountant (Thermo Fisher Scientific) was used as a mounting media. Confocal images of fixed cells were taken using a Confocal White Light Laser (WLL) Leica TCS SP8 Microscope with the HC Plan Apo 40x/1.30 63x/1.40 or 100 x/1.40 OIL (CS2) objective or a Zeiss LSM880 AxioObserver with Plan-Apochromat 40x/1.40. Image acquisition was carried out with the Leica Application Suite X (LAS X) software (Leica Microsystems) or Zen software (Zeiss). Wide-field images of fixed cells (Figure 4C) were taken using the Nikon Eclipse TE2000 Inverted Microscope with the Plan Apo VC 60 x or 100 x/1.40 OIL objective. After acquisition, images were imported into Fiji (Schindelin et al., 2012) to obtain maximum intensity projections of entire z-stacks. Fiji and Photoshop (Adobe, 2017) were used to perform level adjustment. The immunofluorescent image shown in Figure 4A was generated by deconvoluting the original image using Huygens Professional (Scientific Volume Imaging). For *Chlamydomonas* immunofluorescence, ∼10^7^ Chlamydomonas cells were first resuspended in 0.5 ml M-N/5 medium for 4 hours to allow flagellar assembly. Cells were then treated with autolysin for 30 min at 25°C to remove cell walls, followed by resuspension in 1 ml MTSB (microtubule stabilization buffer, 30 mM HEPES, pH 7.4, 5 mM MgSO_4_, 15 mM KCl, 2 mM EGTA) at room temperature. Fifty microliters of cells were applied to a 0.1% poly-L-Lysine (Sigma-Aldrich) coated well on a multi-well slide (Thermo Fisher Scientific) for 2 minutes in the dark. Excess cell suspension was removed by pipetting. Fifty microliters of lysis buffer (MTSB + 1% NP-40) was added to each well to lyse the cells for 2 minutes at room temperature. MTSB was used to wash individual wells once and removed by pipetting. Samples were fixed with MTSB + 4% paraformaldehyde for 30 minutes at room temperature. Excess liquid was removed by pipetting before slides were submerged in cold methanol (−20°C) for 2 × 5 min and left to dry at room temperature. The remained nucleo-flagellar apparatuses attached to the wells were rehydrated with the addition of PBS (phosphate-buffered saline) for 10 minutes at room temperature. After rehydration, the samples were blocked with 100% blocking buffer (5% BSA and 1% fish gelatin in PBS) for 1 hour at room temperature, followed by inoculation with primary antibodies (diluted with 20% blocking buffer) at 4°C overnight. The samples were washed six times with 20% blocking buffer, followed by 1 hour inoculation at room temperature with secondary antibodies diluted with 20% blocking buffer. The samples were washed six times with 20% blocking buffer and mounted in Fluoromount-G (Southern Biotech). The images were captured with an UltraVIEW VoX laser spinning disk confocal microscope (PerkinElmer) and acquired by Volocity software (PerkinElmer).

#### Live Cell Imaging

RPE-1 cells were grown on a chambered cover glass (Grace Bio-Labs) in D-MEM/F-12 without phenol red (Thermo Fisher Scientific) supplied with 10% FBS, and 100 units of penicillin and 100 μg/ml of streptomycin and transfected with GFP-hCCDC61 using Lipofectamine 3000 (Thermo Fisher Scientific). After 24 hours, the media were replaced with the same media with 0.1% (v/v) DMSO or with 5 μg/ml nocodazole, and confocal fluorescent images of GFP positive RPE-1 cells were taken using a Zeiss LSM880 AxioObserver with Plan-Apochromat 40x/1.40, maintained at 37°C, at time points 0, 60, 120, and 180 min.

#### GFP Pulldown Assays

HEK293T cells were grown nearly confluent in 10 cm dishes containing D-MEM glutaMAX (Thremo Fisher Scientific) supplied with 10% FBS. A GFP construct (pEGFP-C1, pEGFP-C1-hCCDC61^1-457; F128E/D129A^ or pEGFP-C1-hCCDC61^1-457; F128E/D129A/5E^) and HA construct (pcDNA3-3xHA-hCCDC61^1-457; F128E/D129A^ or pcDNA3-3xHA-hCCDC61^1-457; F128E/D129A/5E^) were co-transfected into the cells using PEI (Polysciences) as indicated in Figure S4E. The cells were collected after 24 hours of the transfection. GFP-affinity resins were prepared by conjugating purified GFP nanobody to NHS-activated resins (GE Healthcare). The cells were lysed in 1 ml of 50 mM Tris pH 7.4, 200 mM NaCl, 0.2 %(v/v) NP-40, 10%(v/v) glycerol, 1x cOmplete Protease Inhibitor Cocktail EDTA-free on ice for 30 min. Debris was removed by centrifuging at 21,000 g, 4°C for 30 min. 30 μl of the GFP affinity resins were added to the supernatant and incubated on a rotating disk at 4°C for 90 min. The resins were washed five times with the lysis buffer and mixed with 30 μl of 2x SDS sample buffer. Eluted proteins were separated by SDS-PAGE and subject to western blot.

#### Xenopus Embryos

*Xenopus Laevis* embryos culture and injection were carried out as described (Hörmanseder et al., 2017). xCCDC61-RFP (0.1ng), Centrin2-BFP (0.25ng) and Clamp-GFP (0.25ng) mRNAs were injected in one cell stage embryos. Following injection embryos were cultured at 14°C to the tailbud stage (stage 27/28 (Faber and Nieuwkoop, 1994)). Embryos were then fixed for 15 min in MEMFA (100mM MOPS PH6.8, 2mM EGTA, 1mM MgSO4, 4% formaldehyde), washed 3X in 0.1X MBS (MBS (Barth-Hepes Saline) 10X stock : 88 mM NaCl, 1 mM KCl, 2,4 mM NaHCO_3_, 0.82 mM MgSO_4_, 0.33 mM Ca(NO_3_)_2_, 0.41 mM CaCl2, 10 mM HEPES pH 7.4-7.6), and equilibrated overnight at 4°C in 0.1X MBS 50% glycerol. The fixed whole embryos were mounted onto glass slides with ProLong Diamond Antifade Mountant (Thermo Fisher Scientific) and sandwiched with coverslips using a few layers of electrical tape as spacers (Werner and Mitchell, 2013) for confocal imaging.

#### FACS

The day before FACS experiments, 0.5x10^6^ RPE-1 cells were seeded onto each well of a 6-well plate containing 2 ml of the RPE-1 growth media per well. On the next day, Hoechst 33342 (EMP Biotech) was added to the media at the final concentration of 2 μM and incubated at 37°C with 5% CO_2_ for 60 min before the cells were trypsinized and pelleted. The cells were re-suspended in 500 μl of PBS and analysed by flow cytometry using an iCyt EC800 cell analyser (Sony Biotechnology). The resulting cell-cycle distribution of cell singlets was determined using FCS EXPRESS 6 Flow software (*De Novo* Software).

#### Electron Microscopy

For negative staining, 3 μl of sample were applied onto a 400-mesh carbon-coated copper grid (EMS) that was glow discharged and incubated for 1 min at room temperature. After blotting onto filter paper, the grid was washed twice with 5 μl of water and stained with 5 μl of 2%(w/v) uranium acetate for 1 min. The grid was then blotted onto filter paper and air dried. Micrographs were collected using a Tecnai T12 (FEI) operated at 120 kV and equipped with an Orius SC200 or Ultrascan 1000 XP CCD detector (Gatan). Widths of microtubules were measured using Fiji.

### Quantification and Statistical Analysis

We calculated average and standard deviation values using AVERAGE and STDEV functions in Microsoft Excel for Figures 3A, 3E, 4C, S4B, S5B, and S5D, and in GraphPad Prism for Figure S5E. For Figure 5A, χ^2^ analysis was performed using the website (https://wwwsocscistatistics.com/tests/chisquare/). The number of cells with no cilia and the total number of cells were used in pairwise calculations. Sample sizes *n* are provided in figure legends. For the statistical analysis of X-ray crystallography data, details are provided above.

## Supplemental Information

Document S1. Figures S1–S7 and Table S1–S3

Document S2. Article plus Supplemental Information
