# Supplementary material for: CCDC61/VFL3 Is a Paralog of SAS6 and Promotes Ciliary Functions
Source: Structure. 2020 Jun 2;28(6):674–689.e11. doi: 10.1016/j.str.2020.04.010 (PMC7267773; doi:10.1016/j.str.2020.04.010)

Structure, Volume *28*

Supplemental Information

CCDC61/VFL3 Is a Paralog of SAS6

and Promotes Ciliary Functions

Takashi Ochi, Valentina Quarantotti, Huawen Lin, Jerome Jullien, Ivan Rosa e Silva, Francesco Boselli, Deepak D. Barnabas, Christopher M. Johnson, Stephen H. McLaughlin, Stefan M.V. Freund, Andrew N. Blackford, Yuu Kimata, Raymond E. Goldstein, Stephen P. Jackson, Tom L. Blundell, Susan K. Dutcher, Fanni Gergely, and Mark van Breugel

Supplementary tables

Table S1. Related to Figure 1. Accession numbers of selected CCDC61 orthologs.

**Name of organism**		**Accession number**
Homo sapiens	Human	NP_001254652.1
Mus musculus	Mouse	NP_001028486.1
Gallus gallus	Chicken	NP_001006546.1
Xenopus laevis	Frog	XP_018084688.1
Danio rerio	Zebrafish	NP_001070634.1
Capitella teleta	Worm	ELU17212.1
Apis dorsata	Bee	XP_006623417.1
Pediculus humanus corporis	Body louse	XP_002431146.1
Nicrophorus vespilloides	Beetle	XP_017778830.1
Strongylocentrotus purpuratus	Sea urchin	XP_011661626.1
Schmidtea mediterranea	Planaria	SMU15034611
Amphimedon queenslandica	Sponge	XP_019855245.1
Salpingoeca rosetta	Choanoflagellate	XP_004997494.1
Batrachochytrium dendrobatidis JAM81	Fungi	XP_006681050.1
Paramecium tetraurelia strain d4-2	Ciliate	XP_001428378.1
Tetrahymena thermophila SB210	Ciliate	XP_001015880.1
Stylonychia lemnae	Ciliate	CDW82093.1
Thecamonas trahens ATCC 50062	Flagellate	XP_013757734.1
Selaginella moellendorffii	Plant	EFJ09694.1
Chlamydomonas reinhardtii	Green algae	XP_001695308.1
Phytophthora parasitica P1569	Oomycete	ETI35441.1
Trypanosoma cruzi strain CL Brener	Parasite	XP_814690.1
Giardia lamblia P15	Parasite	EFO64425.1
Trichomonas vaginalis G3	Parasite	XP_001298262.1

Table S2. Related to Figure 2, 3 and 4. Primers used for site-directed mutagenesis of human and zebrafish CCDC61 plasmids and human genomic DNA PCR.

Primers	Sequences
hCCDC61$^{F128E/D129A}$-F	TACAGTGTGGAAGAAGCTCGCATTCAT
hCCDC61$^{F128E/D129A}$-R	ATGAATGCGAGCTTCTTCCACACTGTA
hCCDC61^{5E}-F	GAAGCGAGCGAAGAGAGCCTGGAAGCCGAGCTGGAAACCCTGACGAGCGAACTGGCA
hCCDC61^{5E}-R	TTCCAGCTCGGCTTCCAGGCTCTCTTCGCTCGCTTCCGCTTCTTCCAGTTCTTTAGCCA
zCCDC61$^{F129E/D130A}$-F	CACGGTGGAGGAAGCTAGGATACATTA
zCCDC61$^{F129E/D130A}$-R	TAATGTATCCTAGCTTCCTCCACCGTG
zCCDC61^{E5}-F	GAAGCCTCCGAAGAGGCTCTTGAAATAGAGGTTGAAAGTCTGACCACTGAGTTGGCC
zCCDC61^{E5}-R	TTCAACCTCTATTTCAAGAGCCTCTTCGGAGGCTTCAAGTTCCTCTAACTGTTCCACCAG
Human genomic DNA PCR forward	TTCCAGGGTTCCATGGGTCTAGGTTTCTCTCTCATCTCCTT
Human genomic DNA PCR reverse	CGAGGTCGACGAATTCGGCACACTCACAGCCAGCATCGAA

Table S3. Related to Figure 5. Primers used to construct *Chlamydomonas* VFL3 plasmids.

Primers	Sequences
VFL3-1F	GCTGTGCTGGCAGGCTGAAC
VFL3-7R	TGCCCAAAGGCCCAAATGTC
VFL3-NotI-F	CATGCTCGAGCGGCCGCTCGGTCCGATTGGTGCTATG
VFL3-NdeI-R	TCACAGTCTCGCATATGTCGCCCATTTCCTTCACGC
VFL3-13F-AflII	TCAAGAAGTTCCCCGTGTTCGTCA
VFL3-13R-HpaI	CGACAACGGAGTTAACAGAGGCACGGGACGTCCCC
VFL3-14F-HpaI	CCGTGCCTCTGTTAACTCCGTTGTCGGTAAGCTTGTAGCC
VFL3-14R-SalI	CGCTCGCTGGCCGACTC
exon7-HpaI-HA-F	TCCCGTGCCTCTGTTGGCCGCATCTTTTACCCATACG
exon7-HpaI-HA-R	ACCGACAACGGAGTTGCACTGAGCAGCGTAATCTGG
VFL3-7F	TGCTCAGCCGGTTCCTCTTC
VFL3-15R-HpaI	GCATGCATCAGTTAACCGCCTGTGTCGTGCTGGTG
VFL3-15F-HpaI	GACACAGGCGGTTAACTGATGCATGCATGTAGCGGG
VFL3-3R	CACAGATGCACGGTGCCAGA
exon9-HpaI-HA-F	ACGACACAGGCGGTTGGCCGCATCTTTTACCCATACG
exon9-HpaI-HA-R	ATGCATGCATCAGTTGCACTGAGCAGCGTAATCTGG
VFL3-F126D127-F	CTGAGGAGGCCCGCGTGCACTACCCGCTA
VFL3-5R	AGTCCTCCGTCAGCTGCCGT
VFL3-8F	GTGGAGGTGGAGCAGAAGTC
VFL3-F126D127-R	CGCGGGCCTCCTCAGCGGCATAGGTCATAATGAGG
VFL3-5E-F	TCGAGGAGCTCGAGATGGAGATAGAGCAGCTGACGGAGGACTTAGAGGC
VFL3-6R	CCACCACCCGGGAACTTTGA
VFL3-2F	ACCCGCTACCGCTGCTGTTC
VFL3-5E-R	TCTCGAGCTCCTCGATTTGCTCCTCGCAGCGACCCAGCTCGTCC

Supplemental figure legends

<u>Figure S1. Related to Figure 1.</u> Identification of CCDC61 as an XRCC4 superfamily member and sequence alignment of its orthologs. (A) A schematic flow chart of the computational approach used to identify CCDC61 as an XRCC4-superfamily member. (B) Sequence alignment of CCDC61 orthologs from *Homo sapiens*, *Xenopus laevis*, *Danio rerio*, *Schmidtea mediterranea*, *Chlamydomonas reinhardtii* and *Paramecium tetraurelia*. The alignment was generated using the BOXSHADE server. α-helices and β-strands observed in our crystal structures are highlighted with pink and green respectively, and are labelled on top of the alignment. Predicted helices are highlighted with blue boxes. Residue numbers are found next to the corresponding species names. Dark and light blue and red arrows point to residues in the head and coiled-coil domain, respectively, that where mutated in this study to address their functional role in CCDC61. The green arrow points to the position of the nonsense mutation K497X in the CCDC61 ortholog VFL3 in *Chlamydomonas reinhardtii* strain *vfl3-1*.

<u>Figure S2. Related to Figure 2.</u> The N-terminal head domain of CCDC61 homodimerizes in solution. (A) The two head domain interactions of $hCCDC61^{1\text{-}143}$ observed in the asymmetric unit of the corresponding protein crystal. These two different packing interactions of $hCCDC61^{1\text{-}143}$ are shown in the rectangular boxes labelled D1 and D2. The protein chain common between D1 and D2 is represented using the Consurf (Glaser et al., 2003) conservation colour code as defined in the right bottom of the D1 box. (B) Detailed view of the β-zipper found in the head-to-head homodimer interface of $hCCDC61^{1\text{-}143}$ (dimer D1). The location of the β-zipper is indicated by a black square in the overview of the head-to-head homodimer shown on the left. Subunit colouring as in Figure 2A. Hydrogen bonds are represented by dotted lines. (C) AUC sedimentation velocity analysis of $hCCDC61^{1\text{-}143}$ and $hCCDC61^{1\text{-}143;\ F128E/D129A}$ in solution. Upper panels show Rayleigh interference profiles with best fits of a c(s) model (coloured lines) and their residuals to the fits underneath. The different colors represent scans at different times: blue is the earliest time points

where very little material has sedimented; through to red where all the material has sedimented and the signal is near baseline across the radius. For clarity, only every 3rd scan and 7th data point is displayed. The lower panels show the c(s) distribution of species. The wild-type protein had two main sedimenting species: the species at 1.27 S ($S_{w,20}$ = 2.02 S) had a calculated mass of approx. 17.5 kDa with frictional ratio (f/f_o) = 1.20, close to that expected for a monomer (16.5 kDa); and a species at 1.70 S ($S_{w,20}$ = 2.71 S) with a calculated mass of 27.3 kDa close to the mass expected for a dimer (33 kDa). hCCDC61$^{1\text{-}143;\ F128E/D129A}$ had only one main sedimenting species at 1.15 S ($S_{w,20}$ = 1.83 S) with a corresponding mass of approx. 16 kDa with f/f_o = 1.245. This is close to the mass expected for a monomer (16.4 kDa). (D) Analysis of an AUC equilibrium sedimentation experiment with wild-type protein in three different concentrations (1.3, 4 and 12 mg/ml) at 11,600 (dark blue), 19,700 (light blue) and 34,000 rpm (maroon). For clarity only every 6th data point is displayed. The data revealed average molecular masses from 22-33 kDa consistent with a monomer-dimer equilibrium. Fitting such a model to the data gave a global value for the dimerisation dissociation constant K_d = 170 ±18 μM. (E) 2D ^{1}H,^{15}N BEST-Trosy spectra of ^{15}N-labelled human SAS6$^{1\text{-}143}$ alone (black) or mixed with unlabelled hCCDC61$^{1\text{-}143}$ (red). No significant chemical shift perturbation of ^{15}N-labelled human SAS6$^{1\text{-}143}$ was observed in the presence of unlabelled hCCDC61$^{1\text{-}143}$, arguing against an interaction between these two proteins.

Figure S3. Related to Figure 2. Comparison of XRCC4 superfamily proteins. (A) Structural comparison of zCCDC61 (pink; Figure 2C), *L. major* SAS6 (cyan); PDB code: 4CKP (van Breugel et al., 2014)), *C. elegans* SAS6 (sky blue; PDB code: 3PYI (Hilbert et al., 2013)) and *H. sapiens* XRCC4/XLF (yellow and grey respectively; PDB code: 3W03 (Wu et al., 2011);). (B) Superposition of the head domains of zCCDC61 (pink), *L. major* SAS6 structures (cyan), *C. elegans* SAS6 (sky blue) and *H. sapiens* XRCC4/XLF (yellow and grey respectively). Both the relative head-to-head dimer orientations and the head-domain – coiled-coil domain orientation differ between these structures, explaining the different oligomeric assemblies formed by them. The relative orientations

of the head-to-head dimers were measured using Cα atoms of a conserved hydrophobic residue indicated by the red arrowhead in Figure S7A.

<u>Figure S4. Related to Figure 3.</u> CCDC61 binds microtubules. (A) Transiently over-expressed hCCDC61 colocalizes with microtubules in cells. Immunofluorescent images of RPE-1 cells expressing the indicated GFP-hCCDC61 constructs and stained against GFP and α-tubulin. Scale bars are 10 µm. (B) Microtubule-stabilizing and -destabilizing agents do not affect the proportion of cells containing CCDC61 filaments. Immunofluorescent images of RPE-1 cells expressing GFP-hCCDC61 that were treated with DMSO (control), 5 µM taxol (Taxol) or 5 µg/ml nocodazole (Noco.) for 3 hours. The regions indicated by white rectangles (1: CCDC61 containing, 2: largely devoid of CCDC61) are shown magnified in the second and third columns. α-tubulin staining of the regions devoid of CCDC61 indicated that in these regions taxol-treated cells tend to have thicker microtubule bundles whereas nocodazole-treated cells have a more dispersed α-tubulin staining there. Scale bars, 20 µm. Bar graphs show the ratios of cluster-only versus filament-containing RPE-1 cells expressing GFP-hCCDC61 observed under the experimental conditions (DMSO (*n*=169), taxol (*n*=240), nocodazole (*n*=253)). Error bars are standard deviations calculated from three biological replicates. (C) CCDC61 filaments are retained in the presence of nocodazole. Live cell imaging of a control RPE-1 cell after addition of 0.1%(v/v) DMSO and three RPE-1 cells (cell 1: upper panel, cell 2: lower panel with white arrowheads and cell 3: lower panel with white arrows) containing GFP-hCCDC61 filaments after addition of 5 µg/ml nocodazole. Scale bar, 10 µm. (D) Conserved positively charged residues in α7 are essential for microtubule binding by hCCDC61. Coomassie stained SDS-PAGE gel showing a co-pelleting assay with taxol-stabilized microtubules and the 5E mutant of the coiled-coil domain of hCCDC61. S, supernatant, P, pellet. (E) Western blot showing the results of a GFP-pulldown from tissue culture cell extracts of HEK293T cells transiently overexpressing GFP- or 3xHA-tagged hCCDC61 mutants. Shown is the ponceau stained blot (top) as well as the blot staining with an anti-HA antibody (bottom). (F) Circular dichroism (CD) spectra of zCCDC61$^{146\text{-}280}$ (black) and its 5E mutant (red). The figure shows CD

spectra at 5 °C (left) and melting curves of these constructs as observed at 222 nm at increasing temperatures (right). (G) Removal of the C termini of tubulins in microtubules by subtilisin. 2 mg/ml taxol-stabilised microtubules "C" were incubated with a four-fold dilution series of subtilisin. The highest used concentration of subtilisin was 2 mg/ml. Reactions were stopped by PMSF addition and parts of these reactions were separated by SDS-PAGE followed by Coomassie Blue staining. The corresponding gel is shown here. The remainder of the reaction marked by a black arrow was subsequently used for the co-pelleting assay shown in (H), in the panel beneath. (H) The C-terminal tails of tubulins are required for CCDC61 binding to microtubules. Coomassie stained SDS-PAGE gel showing a co-pelleting assay of the coiled-coil domain of hCCDC61 with either untreated taxol-stabilized microtubules (MT) or subtilisin-treated taxol-stabilized microtubules that lack the C-terminal tails of tubulin ($MT^{\Delta C}$, see (G)). S, supernatant, P, pellet. (I) Immunofluorescent images of RPE-1 cells overexpressing GFP-$hCCDC61^{5E}$ and stained against GFP and α-tubulin. We examined a total of 101 cells from three biological replicates but did not observe any filament formation by GFP-$hCCDC61^{5E}$. Scale bar is 20 μm.

Figure S5. Related to Figure 4. CCDC61 does not play a major role in cell division but has a function in ciliogenesis. (A) CRISPR/Cas9 knockout strategy and results of *CCDC61* knock-out in RPE-1 cells. Inserts (pink) and premature-stop codon positions (pink arrows) are indicated in a schematic diagram of the genomic locus of *CCDC61*. An agarose gel image shows genomic PCR results of two-independent *CCDC61* knockout RPE-1 cells. (B) FACS profiles of control and two *CCDC61*-knockout RPE-1 cells. The colour scheme of the FACS profile of each cell is as follows: experimental data, which are cell count x Hoechst-area, (black), diploid in G0/G1 (red "\"), diploid in S (red "|"), diploid in G2/M (red "/") and fitted curve (green). Bar graphs show quantification of the numbers of cells in G1, S and G2/M cells from three biological replicates. Error bars are standard deviations. (C) Centrosome numbers of control and *CCDC61* knockout RPE-1 cells. The numbers of centrioles of monastrol treated RPE-1 cells were counted by immunostaining with Centrin-3. 30 mitotic cells were counted per cell line from one sample per cell type. (D) Proliferation profiles of

control and the *CCDC61*-knockout RPE-1 cells. Data correspond to three biological replicates. Error bars are standard deviations. (E) Reduction of ciliated cells upon knockdown of *CCDC61* in RPE-1 cells. The top bar graphs show knockdown efficiencies of cell only (Cells), transfection-reagent only (RNAiMAX), control siRNA (CT siRNA) and three different siRNAs against *CCDC61* (siRNA 1,2 and 3), assessed by RT-PCR and calculated from three biological replicates. The bottom bar graphs show ciliogenesis efficiencies of these control and CCDC61 knockdown RPE-1 cells. Data correspond to three biological replicates (total cell counts *n*=1130, 1157, 717, 738, 715 and 565 for Cells, RNAiMAX, CT siRNA, siRNA 1, siRNA 2 and siRNA 3 respectively). Percentages are relative to CT siRNA-treated cells. Bar graphs show mean ± standard deviation. Representative immunofluorescent images used for the quantification of the ciliogenesis efficiencies are shown on the right. Scale bar is 10 μm.

Figure S6. Related to Figure 5. Rescue of *Chlamydomonas vfl3* strains. (A) Abnormal striated fibers in *vfl3* strains. Staining of striated fibers in the wild-type, *vfl3-1* and *vfl3-2* mutants, and the rescued *vfl3-2* strains. Striated fibers are indicated by staining of centrin (green) and cilia are indicated by staining of acetylated α-tubulin (red). Scale bar is 4 μm. (B) Expression of mRNA of *VFL3* in the wild-type strain, *vfl3* strains and the strains expressing VFL3 constructs. CNK10 was used for control. (C) Five independent transformants carrying the 3x HA tag in exon 7 of *VFL3*. Molecular weights are indicated on the right. (D) Transformants carrying wild-type, 5E-, and FD-VFL3 all express the VFL3 protein. Molecular weights are indicated on the right.

Figure S7. Related to Figure 1 Comparison of the XRCC4 superfamily members. (A) Structure-guided sequence alignment of the XRCC4 superfamily members. A conserved motif is indicated by a red-dotted rectangle. The red arrowhead indicates the conserved hydrophobic residue whose Cα atoms were used to measure the relative orientations of the head-to-head dimers in Figure S3B. (B) Amino-acid sequence identities between the XRCC4 superfamily members as observed in their N-terminal head domains. The alignment shown in (A) was used to calculate sequence identities

using the SIAS server (http://imed.med.ucm.es/Tools/sias.html). Shown below these values are the R.M.S.D. values of superpositions of the corresponding N-terminal head domain structures. (C) A hypothetical CCDC61 filament bundle. Two linear zCCDC61 filaments (surface representation in cyan and pink) were superimposed onto the CCDC61 D2 dimer (Figure S2A) found in the asymmetric unit of the hCCDC61$^{1\text{-}143}$ crystal (cartoon representation). The two dimers D1 (red and blue) and D2 (red and green) are indicated by black arrows. (D) Coomassie stained SDS-PAGE gels showing the purified hCCDC61$^{1\text{-}143}$, hCCDC61$^{1\text{-}143;\ F128E/D129A}$, zCCDC61$^{1\text{-}168;\ F129E/D130A}$, His$_6$-lypoyl-zCCDC61$^{1\text{-}170}$, His$_6$-lypoyl-zCCDC61$^{1\text{-}170;\ F129E/D130A}$, zCCDC61$^{1\text{-}170}$, zCCDC61$^{146\text{-}280}$, zCCDC61$^{146\text{-}280;\ 5E}$ and hSAS6$^{1\text{-}143}$ used for AUC, CD, crystallography, NMR and SEC-MALS experiments.

Figure S1

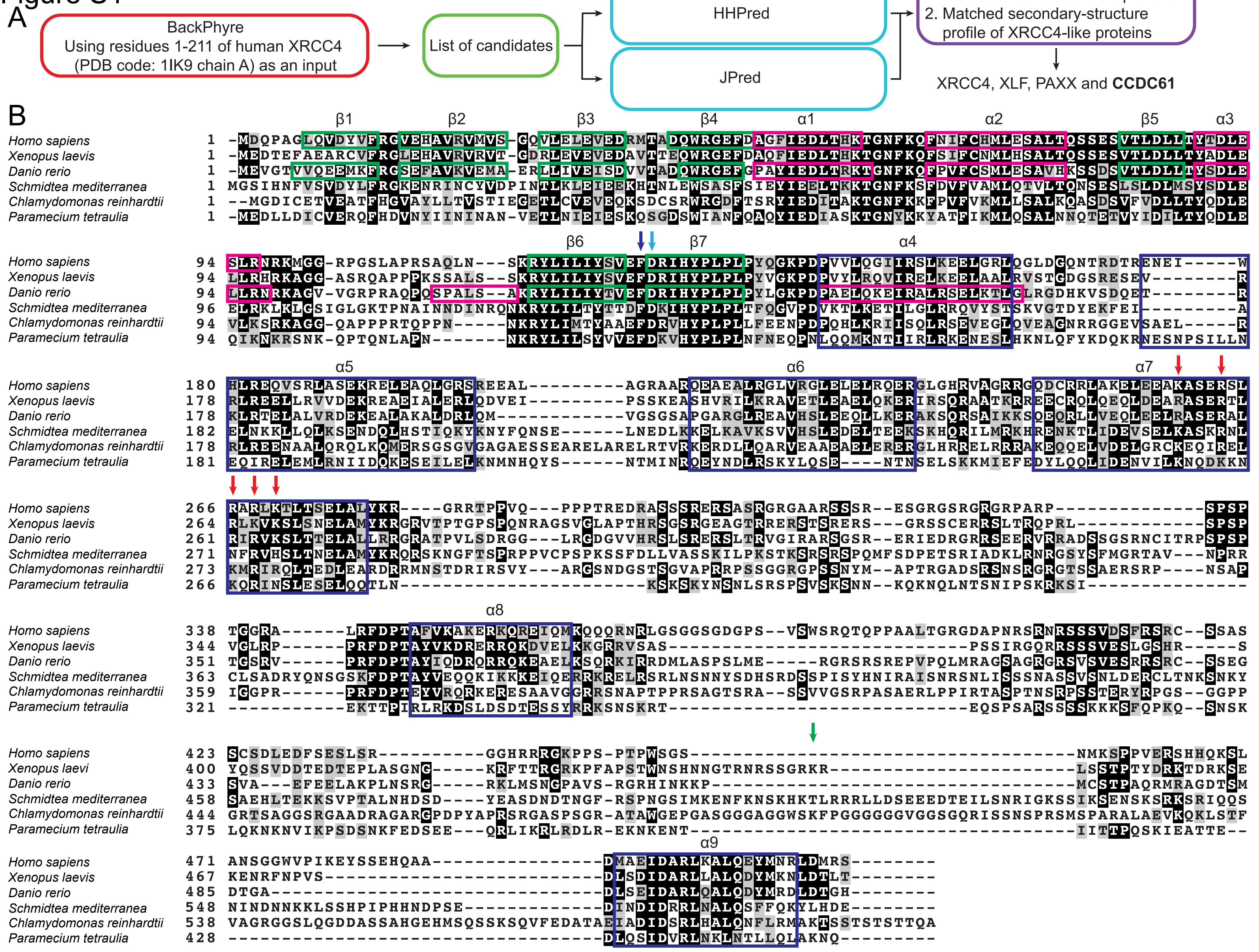
A
BackPhyre
Using residues 1-211 of human XRCC4
(PDB code: 1IK9 chain A) as an input
List of candidates
HHPred
JPred
1. Predicted as XRCC4-like proteins
2. Matched secondary-structure profile of XRCC4-like proteins
XRCC4, XLF, PAXX and CCDC61
B
β1 β2 β3 β4 α1 α2 β5 α3 β6 β7 α4 α5 α6 α7 α8 α9
Homo sapiens
Xenopus laevis
Danio rerio
Schmidtea mediterranea
Chlamydomonas reinhardtii
Paramecium tetraulia
Xenopus laevi

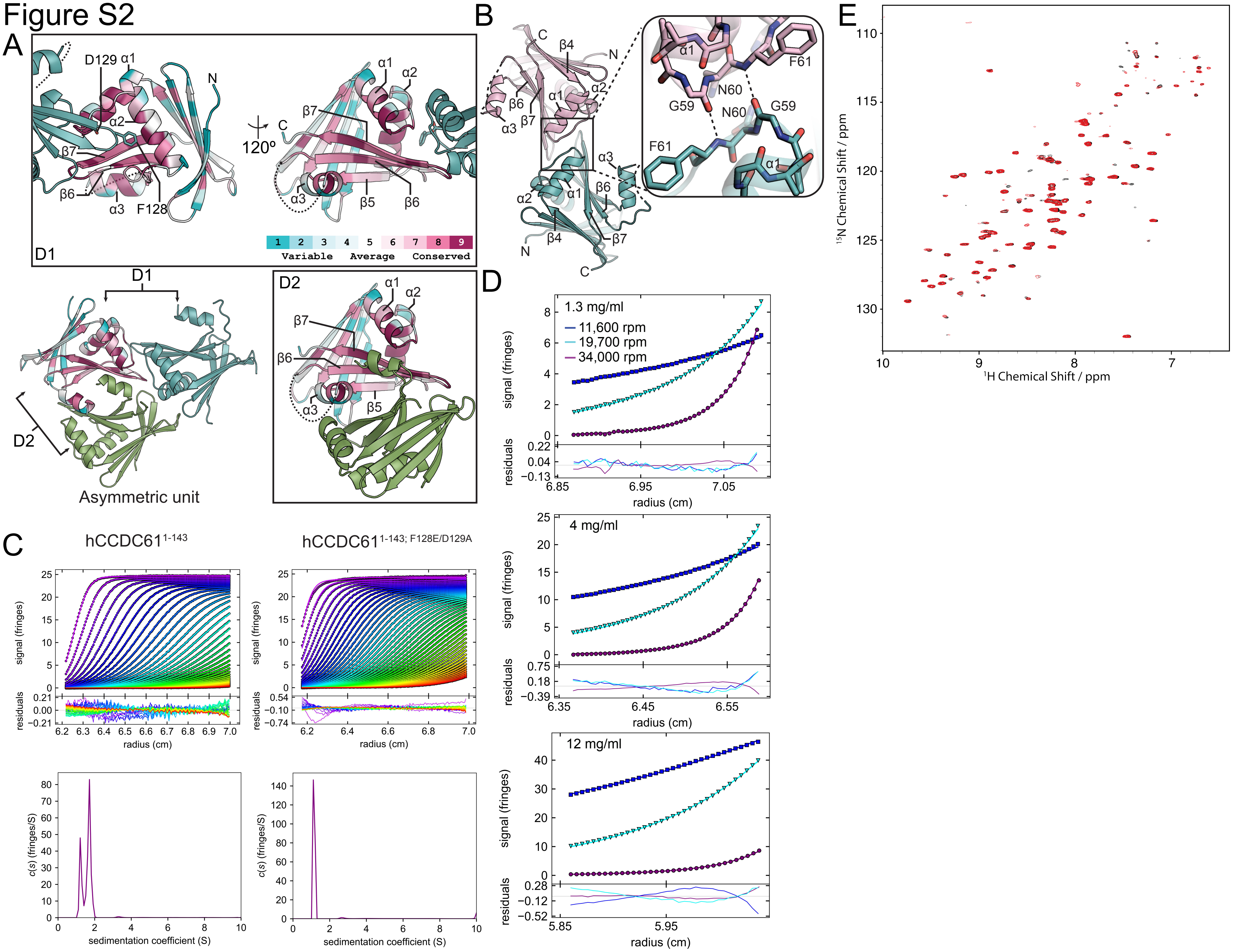
Figure S2
A
D129
α1
α2
β7
β6
α3
F128
N
C
120º
β5
1 2 3 4 5 6 7 8 9
Variable Average Conserved
D1
D2
Asymmetric unit
B
β4
β6
β7
α1
α2
α3
F61
N60
G59
C
D
1.3 mg/ml
11,600 rpm
19,700 rpm
34,000 rpm
4 mg/ml
12 mg/ml
signal (fringes)
residuals
radius (cm)
E
¹⁵N Chemical Shift / ppm
¹H Chemical Shift / ppm
C
hCCDC61¹⁻¹⁴³
hCCDC61¹⁻¹⁴³; F128E/D129A
c(s) (fringes/S)
sedimentation coefficient (S)

Figure S3

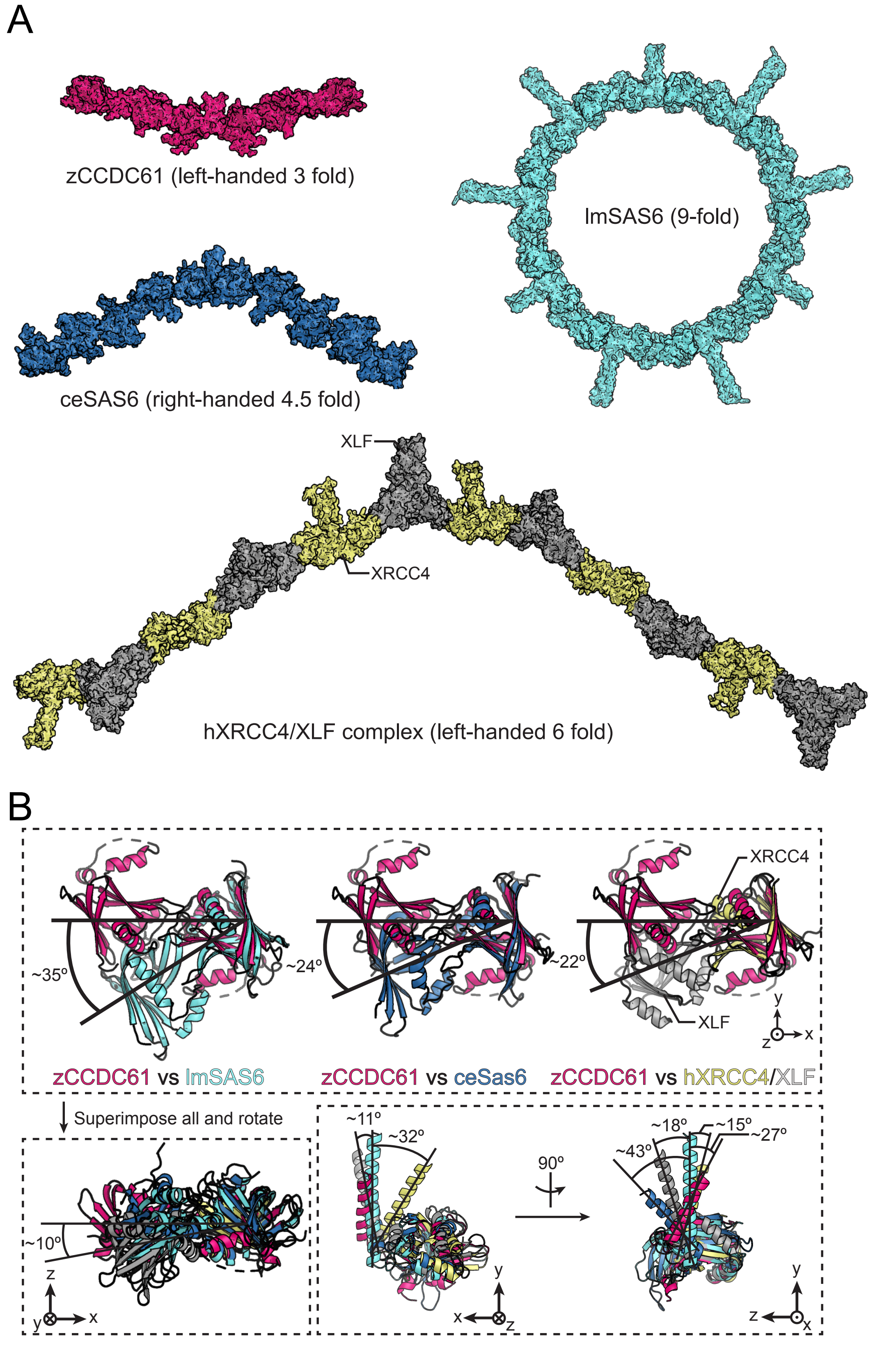
A
zCCDC61 (left-handed 3 fold)
lmSAS6 (9-fold)
ceSAS6 (right-handed 4.5 fold)
XLF
XRCC4
hXRCC4/XLF complex (left-handed 6 fold)
B
XRCC4
~35º
~24º
~22º
XLF
zCCDC61 vs lmSAS6
zCCDC61 vs ceSas6
zCCDC61 vs hXRCC4/XLF
Superimpose all and rotate
~10º
~11º
~32º
90º
~18º
~15º
~27º
~43º

Figure S4

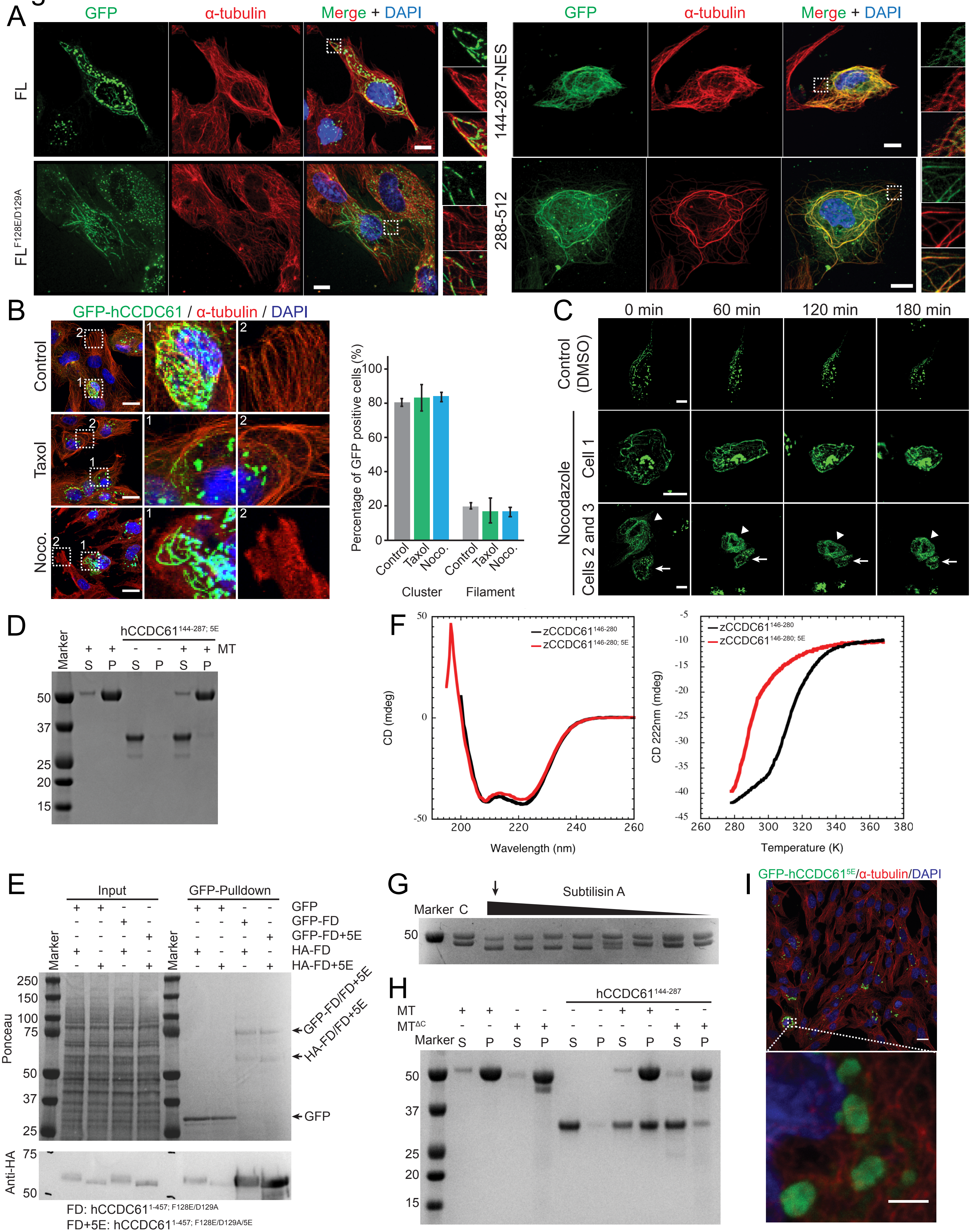
A
GFP
α-tubulin
Merge + DAPI
FL
FL F128E/D129A
144-287-NES
288-512
B
GFP-hCCDC61 / α-tubulin / DAPI
Control
Taxol
Noco.
Percentage of GFP positive cells (%)
Cluster
Filament
C
0 min
60 min
120 min
180 min
Control (DMSO)
Nocodazole
Cell 1
Cells 2 and 3
D
Marker
hCCDC61 144-287; 5E
MT
S
P
E
Input
GFP-Pulldown
GFP
GFP-FD
GFP-FD+5E
HA-FD
HA-FD+5E
Ponceau
Anti-HA
GFP-FD/FD+5E
HA-FD/FD+5E
FD: hCCDC61 1-457; F128E/D129A
FD+5E: hCCDC61 1-457; F128E/D129A/5E
F
zCCDC61 146-280
zCCDC61 146-280; 5E
CD (mdeg)
Wavelength (nm)
CD 222nm (mdeg)
Temperature (K)
G
Marker
C
Subtilisin A
H
hCCDC61 144-287
MT
MTΔC
Marker
I
GFP-hCCDC61 5E/α-tubulin/DAPI

Figure S5

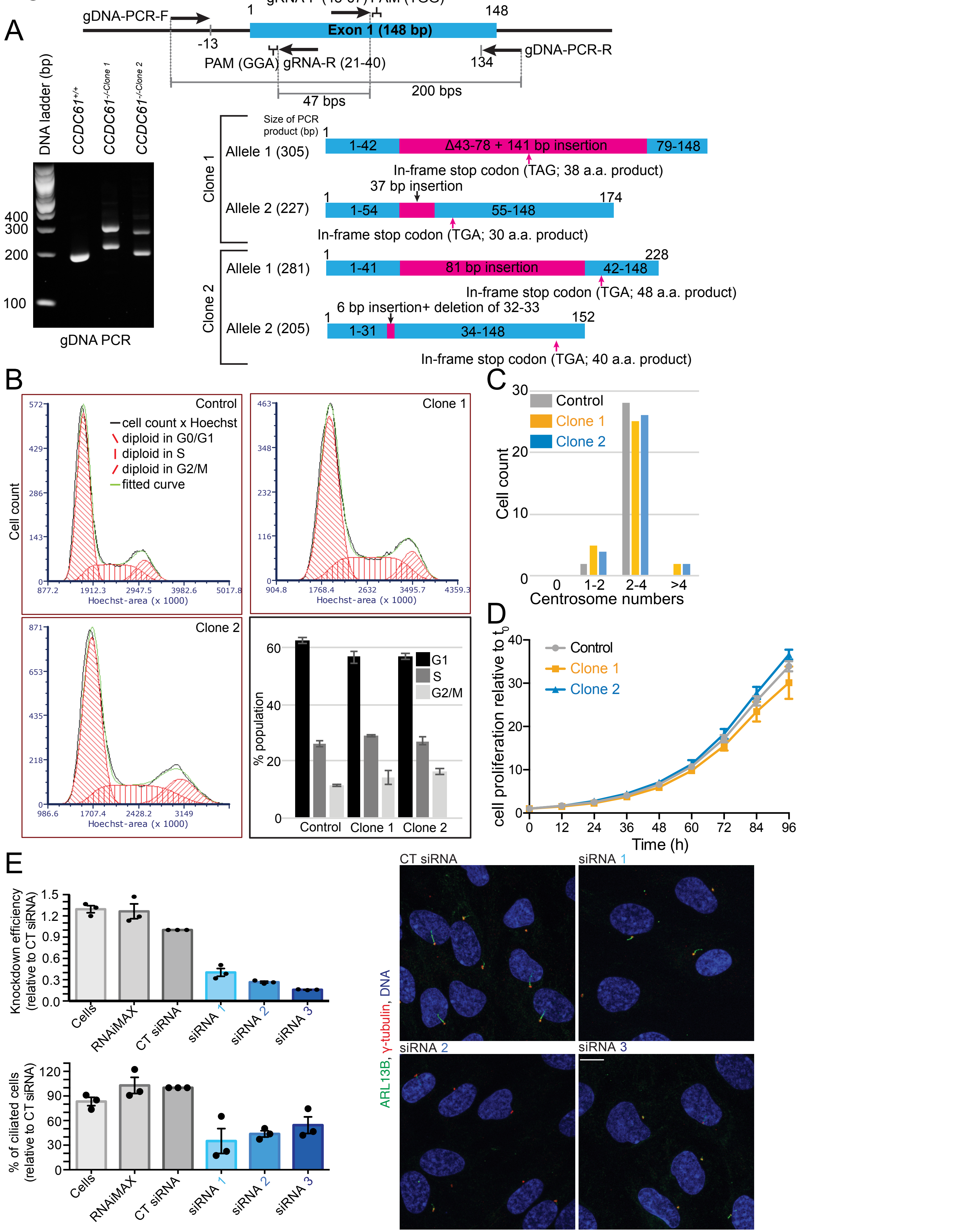
A
gDNA-PCR-F
gRNA-F (48-67) PAM (TGG)
Exon 1 (148 bp)
PAM (GGA) gRNA-R (21-40)
gDNA-PCR-R
47 bps
200 bps
DNA ladder (bp)
gDNA PCR
Clone 1
Clone 2
Allele 1 (305)
Δ43-78 + 141 bp insertion
In-frame stop codon (TAG; 38 a.a. product)
37 bp insertion
Allele 2 (227)
In-frame stop codon (TGA; 30 a.a. product)
Allele 1 (281)
81 bp insertion
In-frame stop codon (TGA; 48 a.a. product)
6 bp insertion+ deletion of 32-33
Allele 2 (205)
In-frame stop codon (TGA; 40 a.a. product)
B
Control
Clone 1
Clone 2
cell count x Hoechst
diploid in G0/G1
diploid in S
diploid in G2/M
fitted curve
Cell count
Hoechst-area (x 1000)
% population
G1
S
G2/M
C
Centrosome numbers
D
cell proliferation relative to t0
Time (h)
E
Knockdown efficiency (relative to CT siRNA)
% of ciliated cells (relative to CT siRNA)
Cells
RNAiMAX
CT siRNA
siRNA 1
siRNA 2
siRNA 3
ARL13B, γ-tubulin, DNA

Figure S6

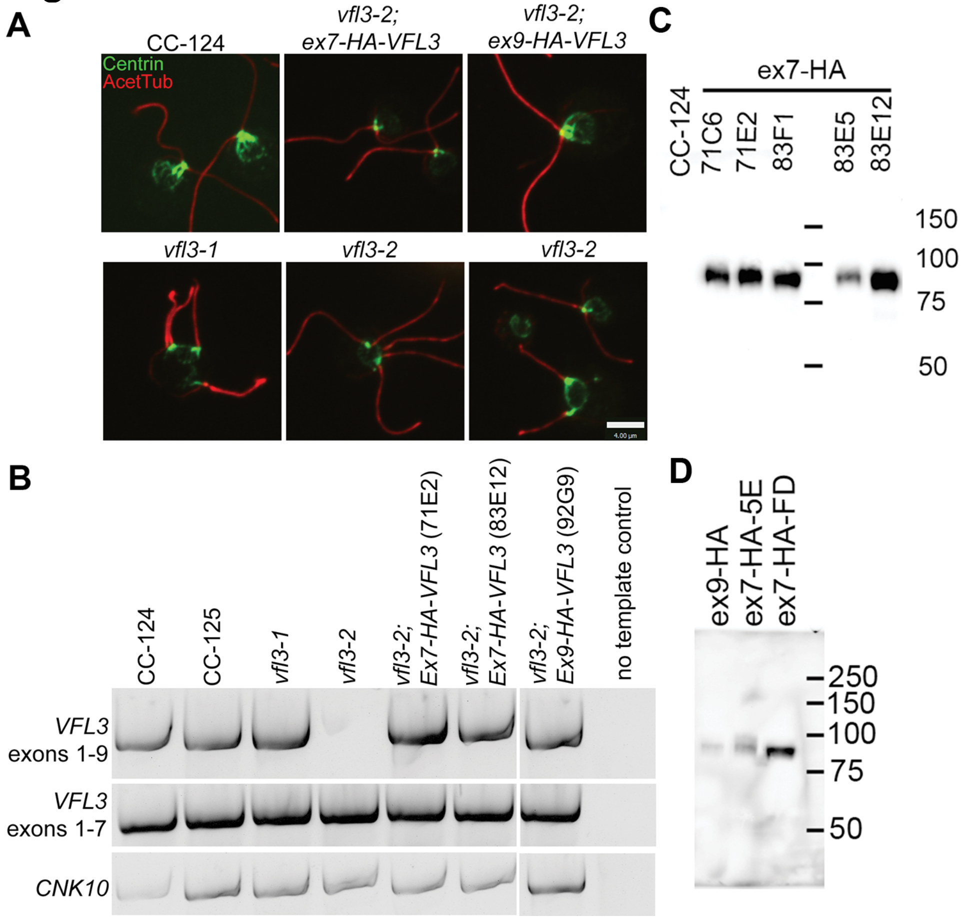
A
CC-124
vfl3-2;
ex7-HA-VFL3
vfl3-2;
ex9-HA-VFL3
Centrin
AcetTub
vfl3-1
vfl3-2
vfl3-2
B
CC-124
CC-125
vfl3-1
vfl3-2
vfl3-2; Ex7-HA-VFL3 (71E2)
vfl3-2; Ex7-HA-VFL3 (83E12)
vfl3-2; Ex9-HA-VFL3 (92G9)
no template control
VFL3 exons 1-9
VFL3 exons 1-7
CNK10
C
ex7-HA
CC-124
71C6
71E2
83F1
83E5
83E12
150
100
75
50
D
ex9-HA
ex7-HA-5E
ex7-HA-FD
250
150
100
75
50

Figure S7

A

```
hCCDC61  1 -----MDQPAGLQVDYVFR---------GVEHAVRVMVSG-------QVLELEVEDRMT-ADQWRGEFDAGFIEDLTHKTGN--FKQFNI
hSAS6    1 ------MSQVLFHQLVPLQVKCKDCEERRVSIRMSIELQSVSNPVHRKDLVIRLTDDTDPFFLYNLVISEEDFQSLKFQQGL--LVDFLA
hXRCC4   1 --------MERKISRIHLV------SEPSITHFLQVSWEK----TLESGFVITLTDG---HSAWTGTVSESEISQEADDMAM----EKGK
hXLF     1 MEELEQGLLMQPWAWLQL---------AENSLLAKVFITK-------QGYALLVSDL---QQVWHEQVDTSVVSQRAKELNKRLTAPPAA
hPAXX    1 ------MDPLSPPLCTLPP------GPEPPRFVCYCEGEE-SGEGDRGGFNLYVTDA---AELWSTCFTPDSLAALKARFGL---SAAED
                                                                        Conserved motif

hCCDC61 67 FCHMLESALTQSS-------ESVTLDLLTYTDLESLRNRKMGGRPGSLAPRSAQLNSKRYLILIYSVEFD-RIHY-PLPLPYQ
hSAS6   83 FPQKFIDLLQQCTQEHAKEIPRFLLQLVSPAA--------------------ILDNSPAFLNVVETNPFKHLTHL-SLKLLPG
hXRCC4  66 YVGELRKALLSGAG----PADVYTFNFS----------------------------KESCYFFFEKNLK-DVSFRLGSFNLEKV
hXLF    72 FLCHLDNLLRPLLKDAAH-PSEATFSCDC------------------------VADALILRVRSELS-GLPFYW-NFHCMLA
hPAXX   72 ITPRFRAACEQ-----------QAVALTLQ----------------------------EDRASLTLS-GGP----SAL-AFDLSKV
```

B

CCDC61	100 / 0.00				
SAS6	12.85 / 4.56	100 / 0.00			
XRCC4	11.20 / 3.06	11.20 / 4.38	100 / 0.00		
XLF	9.52 / 3.37	7.93 / 5.34	15.51 / 4.26	100 / 0.00	
PAXX	13.39 / 4.00	16.96 / 5.93	11.60 / 4.24	8.03 / 3.92	100 / 0.00
	CCDC61	SAS6	XRCC4	XLF	PAXX

Sequence identities (%) / R.M.S.D (Å)

C

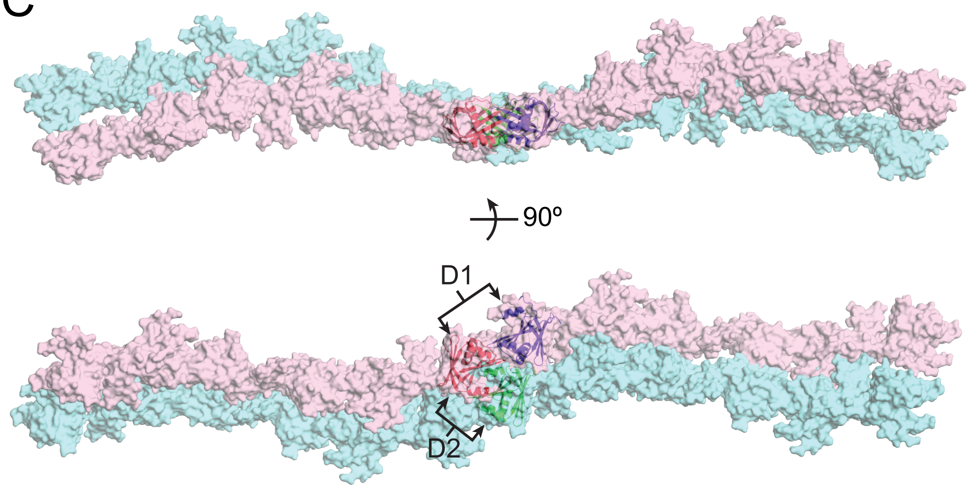

D

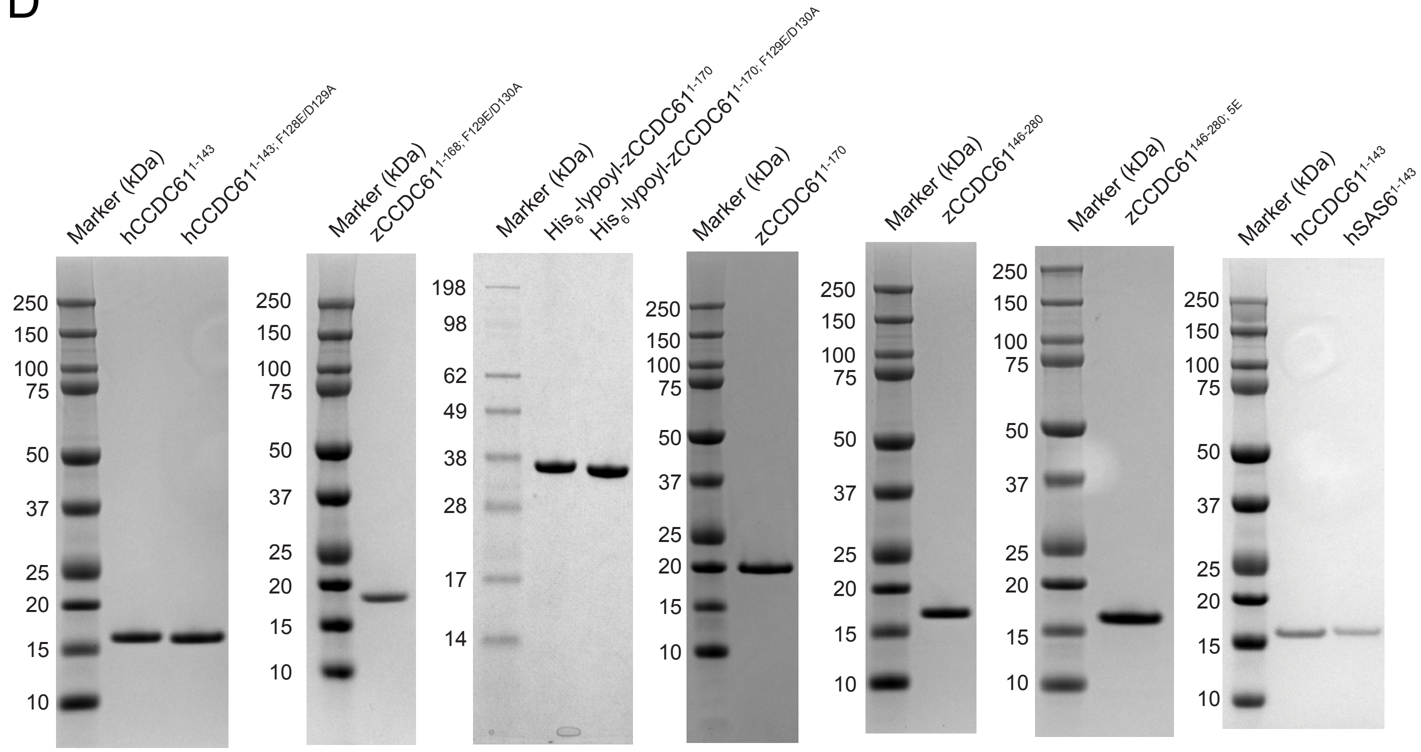

Supplement: Document S1. Figures S1–S7 and Table S1–S3 [file mmc1.pdf]
